# Cyclophostin and Cyclipostins analogues counteract macrolide-induced resistance mediated by erm(41) in *Mycobacterium abscessus*

**DOI:** 10.1186/s12929-024-01091-w

**Published:** 2024-12-03

**Authors:** Morgane Sarrazin, Isabelle Poncin, Patrick Fourquet, Stéphane Audebert, Luc Camoin, Yann Denis, Pierre Santucci, Christopher D. Spilling, Laurent Kremer, Vincent Le Moigne, Jean-Louis Herrmann, Jean-François Cavalier, Stéphane Canaan

**Affiliations:** 1 CNRS, LISM UMR7255, IMM-FR3479, Aix-Marseille Univ, Marseille, France; 2 INSERM, CNRS, Institut Paoli-Calmettes, CRCM, Aix-Marseille Univ, Marseille Protéomique, France; 3 Plateforme Transcriptome, Aix-Marseille Univ, CNRS, IMM-FR3479, Marseille, France; 4 Department of Chemistry and Biochemistry, University of Missouri St. Louis, St. Louis, MO USA; 5 Centre National de la Recherche Scientifique UMR 9004, Institut de Recherche en Infectiologie de Montpellier (IRIM), Université de Montpellier, 34293 Montpellier, France; 6 INSERM, Institut de Recherche en Infectiologie de Montpellier, 34293 Montpellier, France; 7 Université Paris-Saclay, UVSQ, INSERM, Infection et Inflammation, Montigny-le-Bretonneux, France; 8 Assistance Publique-Hôpitaux de Paris, Hôpitaux Universitaires Ile-de-France Ouest, GHU Paris-Saclay, Hôpital Raymond Poincaré, Garches, France

**Keywords:** *Mycobacterium abscessus*, Drug susceptibility, Macrolide, Resistance, Synergy testing, Erm(41)

## Abstract

**Background:**

*Mycobacterium abscessus* is an emerging pathogen causing severe pulmonary infections, particularly in individuals with underlying conditions, such as cystic fibrosis or chronic obstructive pulmonary disease. Macrolides, such as clarithromycin (CLR) or azithromycin (AZM), represent the cornerstone of antibiotherapy against the *M. abscessus* species. However, prolonged exposure to these macrolides can induce of Erm(41)-mediated resistance, limiting their spectrum of activity and leading to therapeutic failure. Therefore, inhibiting Erm(41) could thwart this resistance mechanism to maintain macrolide susceptibility, thus increasing the rate of treatment success. In our previous study, the Erm(41) methyltransferase was identified as a possible target enzyme of Cyclipostins and Cyclophostin compounds (**CyC**).

**Methods:**

Herein, we exploited this feature to evaluate the in vitro activity of CLR and AZM in combination with different **CyC **via the checkerboard assay on macrolide-susceptible and induced macrolide-resistant *M. abscessus* strains selected in vitro following exposure CLR and AZM.

**Results:**

Our results emphasize the use of the **CyC** to prevent/overcome Erm(41)‑induced resistance and to restore macrolide susceptibility.

**Conclusion:**

This work should expand our therapeutic arsenal in the fight against a antibioticresistant mycobacterial species and could provide the opportunity to revisit the therapeutic regimen for combating *M. abscessus* pulmonary infections in patients, and particularly in *erm(41)*-positive strains.

**Supplementary Information:**

The online version contains supplementary material available at 10.1186/s12929-024-01091-w.

## Background

*Mycobacterium abscessus* is a rapid growing mycobacteria (RGM) causing pulmonary diseases in vulnerable individuals, such as cystic fibrosis (CF) or bronchiectasis patients [1–3]. These infections are particularly challenging to cure because *M. abscessus* is intrinsically resistant to most antibiotic classes and anti-tuberculosis drugs [4–8]. The current treatment consists in a multidrug regimen which involves an initial phase comprising a macrolide, usually clarithromycin (CLR) or azithromycin (AZM), associated with other antibiotics such amikacin (AMK), tigecycline, imipenem (IPM) or cefoxitin (CFX) for 3–12 weeks. This initial phase is followed by a continuation phase that comprises a macrolide, amikacin and one or several additional antibiotics (minocycline, clofazimine and moxifloxacin) depending on the severity of the infection, the tolerability of the regimen and the drug susceptibility profile of the strain [2, 9]. Macrolides are important in the context of polychemotherapy against NTMs. Despite this, the success rate of treatments using them against *M. abscessus* remains low (between 30 and 50%) [10, 11], particularly in the case of inducible macrolideresistant strains [12]. Macrolides target the large subunit of the bacterial ribosome and bind/occlude the nascent peptide exit tunnel, therefore inhibiting protein synthesis. However, prolonged exposure to macrolides, even at sub-inhibitory concentrations, can lead to the apparition of an inducible macrolide-resistant phenotype mediated by the Erm(41) methyltransferase [13–17]. This enzyme catalyzes the specific methylation of the adenine 2270 of the 23 S rRNA, which protects the ribosome from the macrolide activity. This mechanism can be observed in vitro, by exposing a susceptible strain to macrolides for up to 14 days instead of the classical 3–5 days [13, 15]. This intrinsic resistance mechanism occurs in *M. abscessus* subspecies *M. abscessus* as well as in *M. abscessus* subspecies *M. bolletii*, both expressing a functional Erm(41) enzyme, unlike *M. abscessus* subspecies *M. massiliense* which harbors a truncated version of the *erm(41)* gene [13, 18]. In this context, it is important to characterize the species within the abscessus complex, for example by using *erm41* PCR, which allows both speciation within the complex and the determination of inducible resistance [9], hence its value as a complement to phenotypic tests [19, 20]. Thus, a yet unmet medical need would consist to develop new therapeutic approaches to counteract Erm(41)-induced resistance in *M. abscessus* [21, 22]. Similar strategies have been set up with the use of β-lactamase inhibitors to render *M. abscessus* strains susceptible to β-lactams [7, 23, 24] and highlight the importance and potential of molecules able to block drug resistance as attractive therapeutic adjuncts to reduce and/or circumvent resistance to antibiotics. Recently, the combination of rifabutin with CLR has been reported to suppress CLR resistance in *erm(41)*-expressing strains [24], whereby rifabutin acts as an inhibitor of *erm(41)* transcriptional induction, maintaining *M. abscessus* in a phenotypically susceptible state to CLR.

Cyclipostins and Cyclophostin analogues (**CyC**) represent attractive molecules with potent antibacterial activity against *M. abscessus in vitro* and inside infected macrophages with very low toxicity toward host cells [25–29]. Among these, the enolphosphate analogs **CyC**_**17**_ and **CyC**_**31**_ were the most active growth inhibitors of *M. abscessus* (against both smooth and rough variants) sharing minimal inhibitory concentrations (MIC) similar to those of imipenem or cefoxitin, often used in clinical settings [28, 29]. **CyC** compounds react with enzymes containing a catalytic serine and/or cysteine in their active site by forming an irreversible covalent bond [30–33]. This characteristic was exploited to identify their target enzymes using a competitive activity-based protein profiling (ABPP) approach [26–28, 30, 32–35]. In *M. abscessus*, 39 potential target enzymes of **CyC**_**17**_ were identified, most of which playing a role in lipid metabolism or cell wall synthesis [26]. In addition, 9 out of 39 have orthologs annotated as essential in the *Mycobacterium tuberculosis* genome [26]. Interestingly, 7 methyltransferases were also identified, including Erm(41). This finding highlights the potential of the **CyC** to thwart inducible macrolide resistance by blocking Erm(41), opening the possibility of exploiting these inhibitors as adjuncts molecules to suppress macrolide inducible resistance in the context of anti-*M. abscessus* chemotherapy.

In this study, we validated Erm(41) as an effective target of the **CyC** analogues and investigated their synergistic activity when given in combination with macrolides (CLR or AZM) as well as with other commonly used antibiotics (AMK, CFX and IPM) in susceptible and induced macrolide-resistant *M. abscessus* strains. Our results emphasize the potency of the **CyC** to restore the susceptibility of strains to macrolides, expanding our therapeutic arsenal against *M. abscessus* pulmonary diseases.

## Methods

### Antibiotics and compounds

Clarithromycin (Euromedex, France) and Azithromycin (Sigma Aldrich) were solubilized in 96% ethanol and dimethyl sulfoxide (DMSO), respectively. AMK, IPM and CFX was from Toku-E (France) and dissolved in water. The **CyC** analogues **CyC**_**17**_, **CyC**_**31**,_**CyC**_**8α**_ and **CyC**_**8β**_ were synthesized as described previously [32, 36]. Stock solutions (10 mM in DMSO) of the **CyC** compounds (purity of ≥ 95%) [27, 29] were stored at 4 °C.

### Strain and bacterial culture

*Escherichia coli* DH10B cells used in cloning experiments were grown in Luria-Bertani broth (Invitrogen, Carlsbad CA, USA) or on agar plates at 37 °C. Transformants were selected on LB agar supplemented with 200 µg/mL hygromycin B (Toku-E). *Mycobacterium smegmatis* mc^2^155 *groEL1ΔC* strain [37] was grown in Middlebrook 7H9 (BD Difco) supplemented with 0.05% Tween 80 (Sigma-Aldrich, Saint-Quentin Fallavier, France) and 0.2% glycerol (Euromedex, France) (7H9-S) at 37 °C. For the *M. abscessus* CIP104536^T^ [38] and *M. massiliense* (CIP 108297^T^) [39] S morphotypes, 7H9 was supplemented with 0.05% Tween 80, 0.2% glycerol and with 10% Oleic acid Albumin Dextrose Catalase (OADC) enrichment (BD Difco) (7H9-S^OADC^).

### Expression and purification of recombinant Erm(41)

The *MAB_2297* gene encoding Erm(41), was amplified by PCR using *M. abscessus* genomic DNA and the forward primer 5′GTATAACCATGGTTTCCGGCCAACGGTCGCGAC-3′ (NcoI site in bold) and reverse primer 5′-CATATTAAGCTTTGCGCCGCCTGATCACCAG-3′ (HindIII site in bold). The PCR product was cloned into the acetamide-inducible pMyC vector digested with NcoI and HindIII, as previously described [40], enabling the incorporation of a polyhistidine tag at the C-terminus of Erm(41). The integrity of the insert was confirmed by DNA sequencing (Eurofins Genomics). Approximately 200 ng of pMyC-*erm(41)* were electroporated into *M. smegmatis* mc^2^155 *groEL1ΔC* competent cells by using a Gene Pulser Xcell™ Electroporation System (BioRad, Marnes-la-Coquette, France) at 2500 V, 25 µF and 600 Ω. Recombinant clones were selected on 7H10 agar plates and used to inoculate 10 mL of 7H9-S supplemented with 50 µg/mL hygromycin at 37 °C under shaking at 180 rpm. After 3 days, the preparation was used to inoculate 2 L of culture medium for a large-scale production and the bacteria were grown at 37 °C under shaking until OD_600nm_ value reached 1.5–2. Production of recombinant proteins was induced by adding acetamide (Sigma-Aldrich) to a final concentration of 0.5% (*w/v*) for 16 h at 37 °C. Cultures were harvested by centrifugation at 4500×*g* for 30 min at 4 °C. The bacterial pellets were resuspended in 30 mL CHES buffer (100 mM N-cyclohexyl-2-aminoethanesulfonic acid, pH 10, 150 mM NaCl and 1% Nlauroylsarcosine) (buffer A) and lysed using three passages through a French Press (Aminco, Silver Spring, MD, USA) at 1100 PSI. The supernatant was centrifuged for 30 min at 17,000×*g* and loaded onto a Ni^2+^-nitrilotriacetic acid (NTA) agarose gel column (Amersham Biosciences, UK). The column was extensively washed with buffer A to remove unspecific proteins and Erm(41) was eluted with buffer A containing 50 mM imidazole. Eluted fractions were collected and analyzed by SDS-PAGE followed by dialysis overnight against a solution of 100 mM CHES, pH 10, 150 mM NaCl and 0.05% Nlauroylsarcosine (buffer B) to remove imidazole. The final concentration of the recombinant protein was measured by determination of OD_280nm_ using the molar extinction coefficient (ε = 32290 cm^−1^ M^−1^), concentrated by ultrafiltration to a final concentration of 1.5 mg/mL and stored at −80 °C.

### CyC and Erm(41) interaction

To validate the inhibitory interaction between **CyC** analogues and Erm(41), 10 µg of Erm(41) in 50 µL was incubated for 30 min with different **CyC** inhibitors at a molar excess *x*_*I*_ of 100 in buffer B at 37 °C with shaking. Next, Erm(41) pretreated or not with the **CyC** analogues were further incubated with 5 µM ActivX TAMRA-FP probe (Thermo Fisher Scientific) for 1 h at room temperature in the darkness. The reaction was stopped by adding 5X Laemmli reducing buffer followed by boiling and proteins were separated by 12% SDS-PAGE. Subsequently, TAMRA FP-labeled proteins were detected by fluorescent gel scanning (TAMRA: λ_ex_ 557 nm, λ_em_ 583 nm) using the Cy^®^3 filter of a ChemiDoc MP Imager (Bio-Rad) before staining the gels with Coomassie Brilliant Blue dye. Densitometric analyses were performed using the ImageLab™ software version 5.0 (Bio-Rad) to determine the fluorescence intensity content per sample. All raw data were exported as CSV files, imported in the R studio software (The R Project for Statistical Computing, version 4.2.1) and graphs were plotted with the ggplot2 package (version 3.4.0). Histogram represented the mean ± standard deviation of two independent replicates.

### Molecular docking in silico

The 3D models structures of Erm(41) (UniProt accession no. B1MAV7) were built with the Phyre2 web portal for protein modeling, prediction and analysis using Erm(E) structure from *Saccharopolyspora erythraea* (PDB 6NVM) and Erm(38) structure from *M. smegmatis* (PDB 7F8B) as structural templates [41]. The flexible side chain method, where the ligand (i.e., **CyC**_**17**_) is joined in an arbitrary conformation with the target [i.e., Erm(41) protein] and then modeled as a fully flexible side chain in the AutoDock/Vina simulation, was used [42].

The PyMOL Molecular Graphics System (version 1.4, Schrödinger, LLC) was used as working environment with an in-house version of the AutoDock/Vina PyMOL plugin to perform in silico molecular docking with the **CyC**_**17**_ [43, 44]. A model structure file was generated for the **CyC**_**17**_ molecule, and its geometry was refined using the Avogadro open-source program (version 1.1.1. “http://avogadro.openmolecules.net/”). The box size used for the receptor was chosen to fit the whole protein and to allow nonconstructive binding positions, and was further refined to the inhibitor binding site in Erm(41).

### Mass spectrometry

Global mass analyses were determined on purified Erm(41) (15 mg/ml in 100 mM CHES, pH 10, 150 mM NaCl and 0.05% Nlauroylsarcosine buffer) pre-incubated or not with **CyC**_**17**_ at a molar excess, *x*_*I*_, of 100. Ten µL of each protein sample ± **CyC**_**17**_ were desalted on ZipTip C4 (Millipore, Molsheim, France) and eluted by 3 µL of sinapinic acid matrix solution in 0.3% TFA/CH3CN (50:50 v/v). One µL of the latter sample was analyzed on a MALDI TOF-TOF Bruker Ultraflex III spectrometer (Bruker Daltonics, Wissembourg, France) controlled by the Flexcontrol 3.0 package (Build 51). This instrument was used at a maximum accelerating potential of 25 kV and was operated in linear mode using the *m/z* range from 20,000 to 100,000 (LP_66kDa_method) as already described [30].

### Erm(41) quantification

Proteomic studies on macrolide-resistant strains were performed from 20 mL of *M. abscessus* cultures. Cells were washed twice in PBS (Phosphate Buffer Saline) solution pH 7 and lysed by mechanical disruption on a BioSpec Beadbeater in PBS containing 8 M urea. Quantification of Erm(41) was performed using Liquid chromatography-mass spectrometry (LC-MS/MS) analysis using an Orbitrap Fusion Lumos Tribrid Mass Spectrometer (ThermoFisher Scientific, San Jose, CA) online with an Ultimate 3000RSLCnano chromatography system (ThermoFisher Scientific, Sunnyvale, CA). MS was performed using a data-independent acquisition (DIA) mode. Quantification was based on relative label-free intensity (LFQ) calculated using the DIA-NN 1.8 algorithm [45]. Main DIA-NN output file was further filtered and the LFQ intensity was calculated using our DIAgui package at 1% q value (https://github.com/marseille-proteomique/DIAgui) [46]. Relative quantification was calculated using fold changes of LFQ intensity under CLR or AZM exposure relative to LFQ intensity in non-exposure condition.

The full mass spectrometry proteomics data have been deposited to the ProteomeXchange Consortium (www.proteomexchange.org) *via* the PRIDE partner repository (https://www.ebi.ac.uk/pride/login) with the dataset identifiers PXD055560.

### Preparation of anti-Erm(41) immune serum

Mouse anti-Erm(41) antibodies were produced as follows. A His-tagged version of Erm(41) was prepared as described above. Purified Erm(41) was subcutaneously injected into five BALB/c mice (Janvier, France) (20 µg per mouse) with incomplete Freund’s adjuvant (1/1, v/v) on days 1, 28, and 57. One week after days 28 and 57, blood samples were obtained from the retroorbital plexus, centrifuged and stored at −20 °C until use. Mouse experiments were performed according to institutional and national ethical guidelines (Agreement n˚783223; approved by the Ministry of Higher Education and Research with APAFIS#11465-2016111417574906v4).

### Synergistic assay

Synergistic activities were tested in 96-well microplates by the checkerboard assay in Middlebrook 7H9-S^OADC^ broth and using microdilution method [47]. MIC of the antibiotics were determined in 96 well flat-bottom Nunclon Delta Surface microplates with lid (Thermo-Fisher Scientific, ref. 167008) using optical density to assess growth. Log-phase bacteria were diluted to a cell density of 5 × 10^6^ cells/mL in 7H9-S^OADC^ medium. Then, 100 µL of this bacterial suspension (5 × 10^5^ cells final per well) was added to each well containing 100 µL of the 7H9-S^OADC^ medium, serial two-fold dilutions of the selected antibiotics or controls to a final volume of 200 µL. In addition, wells containing 200 µL of 7H9-S^OADC^ medium only were used as sterility/background controls, whereas wells containing 100 µg/mL kanamycin (Euromedex) was used as positive control for growth inhibition. Plates were incubated at 37 °C for 4 to 5 days. The optical density (OD) at 600 nm was quantified using a Tecan Spark 10 M™ multimode microplate reader (Tecan Group Ltd., France). Relative growth units were defined as: RGU% = (test well OD_600nm_/mean OD_600nm_ of growth control wells) ×100. For each condition, three biological replicates were performed. The MIC of each drug was defined as the lowest drug concentration that inhibited more than 90% of the bacterial growth.

The fractional inhibitory concentration index (FICI) is the reference parameter for quantifying the interaction between two antibiotics in a combination [48]. The fractional inhibitory concentration (FIC) of antibiotic A is defined as the MIC of antibiotic A in the combination divided by the MIC of antibiotic A alone, and vice versa for the FIC of antibiotic B. The FICI value was obtained by the sum of the FIC_A_ and FIC_B_. A FICI value of less than 0.5 is considered synergistic; between 0.5 and 4, the effect is considered indifferent; when greater than 4, the interaction is antagonistic [48]. All FICI presented correspond to the minimal FICI obtained. All the results were exported as CSV files, imported in the R studio software and graphs were plotted with the ggplot2 package.

### Induction of macrolide resistance in *M. abscessus *strains

Macrolide resistance was induced in the *M. abscessus* S reference strain following exposure to CLR or AZM for 14 days. Briefly, *M. abscessus* S strain was used to inoculate 10 mL of 7H9S^OADC^ at an OD_600 nm_ of 0.05 in absence/presence of 0.1 µg/mL CLR or 0.5 µg/mL AZM corresponding to values ~ 10 times lower than the respective MICs. After 5 days of incubation at 37 °C with shaking (50 rpm), 0.2 mL of the bacterial suspensions were used to inoculate 10 mL of fresh culture medium with or without CLR/AZM, and after 5 additional days of incubation, 0.2 mL of the latter preparation was used to re-inoculate 10 mL of fresh culture medium with or without CLR/AZM. The MIC of CLR or AZM was determined before macrolide exposure and during the induction phase at 5, 10, and 14 days. Susceptibility testing was performed in Middlebrook 7H9-S^OADC^ broth using the microdilution method. MIC of CLR and AZM were determined in 96-well flat-bottom Nunclon Delta Surface microplates with lid using the same method, as described in the previous section. The MIC of each drug was defined as the lowest drug concentration that inhibited more than 90% of the bacterial growth. For each condition, three biological replicates were performed.

### RNA extraction and reverse transcription

RNA was prepared from 10^9^*M. abscessus* cells at different time points 1, 2, 5, 6, 7, 10, 11, 12 and 14 days. The bacteria were harvested and frozen at −80 °C beforehand. Pellet was resuspended in homogenization solution supplemented of 2.5% 1-Thioglycerol (*v*/*v*) and then lysed by mechanical disruption on a BioSpec Beadbeater. Total RNA was purified using Maxwell^®^ 16 miRNA Tissue Kit (Promega) according to the manufacturer’s instructions with an extra TURBO DNase (Invitrogen) digestion step to eliminate the contaminating DNA. Finally, the RNA quality was assessed by Tapestation system (Agilent). To obtain cDNAs, GoScript™ Reverse Transcription System protocol (Promega) was used. The final mix contained 1 µg total RNA, 0.5 µg random primers (Promega), 4 µL GoScript™ 5X Reaction Buffer (Promega), 2 µL of 25 mM MgCl_2_, 1 µL 40 mM dNTP and 1 µL GoScript™ Reverse Transcriptase (Promega).

### Quantitative PCR analysis

Quantitative real-time PCR (qPCR) analyses were performed on a CFX96 real-time system (Bio-Rad). The reaction volume was 15 µL, and the final concentration of each primer was 0.5 µM. Specific primers used for qPCR are the following: e*rm(41)* forward 5′CTCAGGGGAGTTCGTTGTGG-3′ and reverse 5′-CCGCTATCCGGACATCTTCC-3′ and *rrs* (*MAB_r5051*) forward 5′-CATGGTGAGTGGTGCAAAGC-3′ and reverse 5′AGTCTGGGCCGTATCTCAGT-3′. The cycling parameters of the qPCR were 98 °C for 2 min, followed by 45 cycles of 98 °C for 5 s, 64 °C for 10 s, and 72 °C for 1 s. A final melting curve from 65 °C to 95 °C was added to determine the specificity of the amplification. To determine the amplification kinetics of each product, the fluorescence derived from the incorporation of SYBERGreen into the double-stranded PCR products was measured at the end of each cycle using the Sso Advanced Universal SYBRGreen Supermix kit (Bio-Rad, France). The results were analyzed using Bio-Rad CFX Maestro software, version 2.3 (Bio-Rad, France). The 16 S RNA gene (*rrs*) was used as a reference for normalization. For each point, a technical duplicate was performed. All the results were exported as CSV files, imported in the R studio software and graphs were plotted with the ggplot2 package.

## Results

### Characterization of the CyC-Erm(41) interaction

**Figure. 1:**
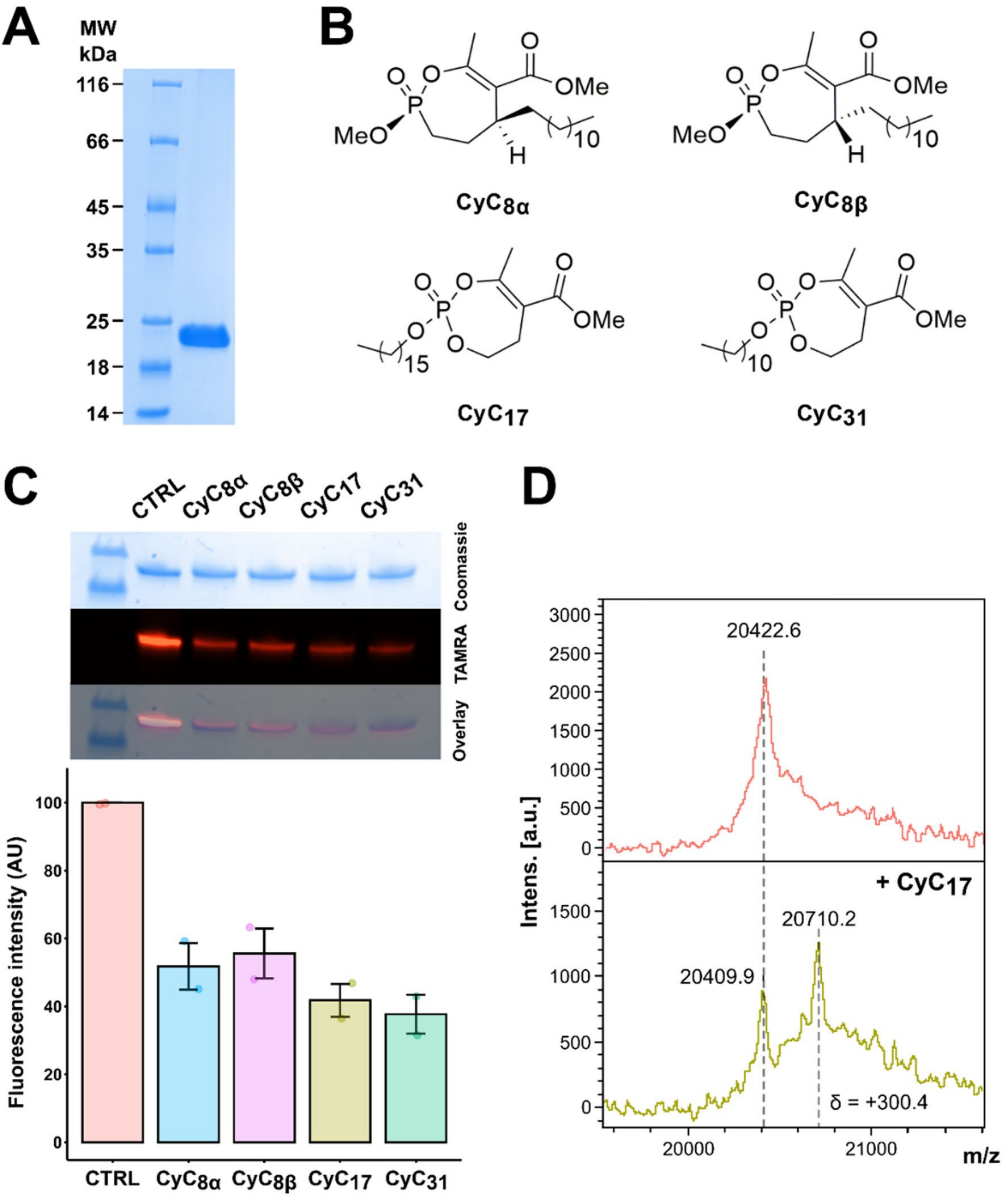
Biochemical characterization of the CyC-Erm(41) interaction. **A** Protein purity assessed by SDS-PAGE. Ten micrograms of protein were loaded onto a 12% polyacrylamide gel and stained with Coomassie brilliant blue G-250 solution. MW, molecular weight standards (5 µg; Euromedex). **B** Chemical structures of the **CyC** analogues used in this study. **C** Equal amounts of Erm(41) were pre-treated with **CyC**_**8α**_. **CyC**_**8β**_. **CyC**_**17**_ and **CyC**_**31**_ , incubated with TAMRA-FP, separated by SDS-PAGE, and visualized by Coomassie Blue staining (top) or in-gel fluorescence (middle). Fluorescence intensity was quantified using the ImageLab™ software. The TAMRA-FP without CyC (CTRL) was arbitrarily placed at 100 AU of fluorescence intensity. D Global mass modification of Erm(41) alone (upper panel) or pre-incubated with CyC **17** (lower panel) as determined using an Ultraflex III mass spectrometer (Bruker Daltonics) in linear mode with the LP_66kDa method

In a previous study, competitive ABPP conducted with the **CyC**_**17**_ inhibitor identified the Erm(41) methyltransferase responsible for inducible macrolide resistance as a possible biological target in *M. abscessus* at a permutation false discovery rate of 5% [26]. To investigate whether other **CyC** analogues displaying activity against *M. abscessus* interact and inhibit Erm(41), the *erm(41)* gene (*MAB_2297*) was cloned within a homemade pMyC vector in frame with a 6xHis-tag, allowing strong expression of Erm(41)-His_6_ in the presence of acetamide [49]. The recombinant protein (theoretical mass ~ 22 400 Da) was produced in *M. smegmatis* mc^2^155 *groEL1ΔC*, and purified to homogeneity by nickel affinity, leading to around 20 mg of pure recombinant protein per liter of culture. Purity and the molecular weight were checked by 12% SDS-PAGE and global mass spectrometry (Fig. 1).

Considering the structure and mechanism of action of the **CyC** analogues on catalytic serine or cysteine residues, a chemically relevant fluorophosphonate (FP) probe bearing a fluorophore (i.e., rhodamine for TAMRA-FP) and exhibiting a similar mode of action [33, 50] was selected and tested in competitive inhibition tests to assess the covalent interaction between Erm(41) and various **CyC** (Fig. 1B). Purified Erm(41) was first incubated with either **CyC**_**17**_ and **CyC**_**31**_, two potent inhibitors of extracellularly-growing *M. abscessus*; or **CyC**_**8α**_ and **CyC**_**8β**_, only active against intracellularly-growing *M. abscessus* [26, 28, 29]. The Erm(41)-CyC complexes were further co-incubated for 1 h with TAMRA-FP and equal amounts of protein were separated by SDS-PAGE and visualized by Coomassie staining (Fig. 1C, upper panel) and ingel fluorescence for TAMRA detection (Fig. 1C, middle panel). In each case, pre-treatment with the **CyC** molecules resulted in a significant decrease in the fluorescence intensity vs. the control, indicating that the reaction with the TAMRA probe was impeded in the presence of Erm(41)-**CyC** adducts. Comparison of the fluorescence intensity between the four **CyC**-Erm(41) complexes and the control condition without **CyC**, showed a decrease in fluorescence of ~ 50–60% (Fig. 1C). Surprisingly, incubation with higher concentrations of CyC, up to a molar excess *x*_*I*_ of 200 related to 1 mol of Erm(41), did not further reduce the fluorescence intensity (data not shown). Taken together, these results suggest that **CyC**_**8α**_, **CyC**_**8β**_, **CyC**_**17**_ and **CyC**_**31**_ directly interact with Erm(41), confirming that this methyltransferase is an effective target of these inhibitors. The fact that the **CyC**-mediated fluorescence inhibition of was incomplete, remains however unexplained.

To further confirm this interaction, the **CyC**_**17**_-Erm(41) complex obtained above was subjected to MALDI-TOF mass spectrometry (Fig. 1D), which allows to track mass changes if the **CyC** inhibitor is covalently bound to the protein. Total mass results showed the presence of two peaks: a first peak (20,409.9 ± 3 Da) corresponds to the unmodified protein, and a second one (20,710 Da) to the **CyC**_**17**_-bound Erm(41) adduct. It is noteworthy, however, that the observed 300.4-Da mass shift increment in global mass was 146.08 Da lower than the expected **CyC**_**17**_ theoretical molecular mass of 446.28 Da (Fig. 1D). This mass difference is consistent with previous studies with the thioesterase TesA [31], the antigen 85 complex [33] and the hydrolase HsaD [30] where rearrangement of the covalently bound **CyC**_**17**_ inhibitors occurred, resulting in the loss of the methyl 2-acetyl-4-hydroxybutanoate moiety to reach a stable thermodynamic state. Overall, these results therefore support the formation of a covalent and irreversible Erm(41)-**CyC** complex.

**Fig. 2:**
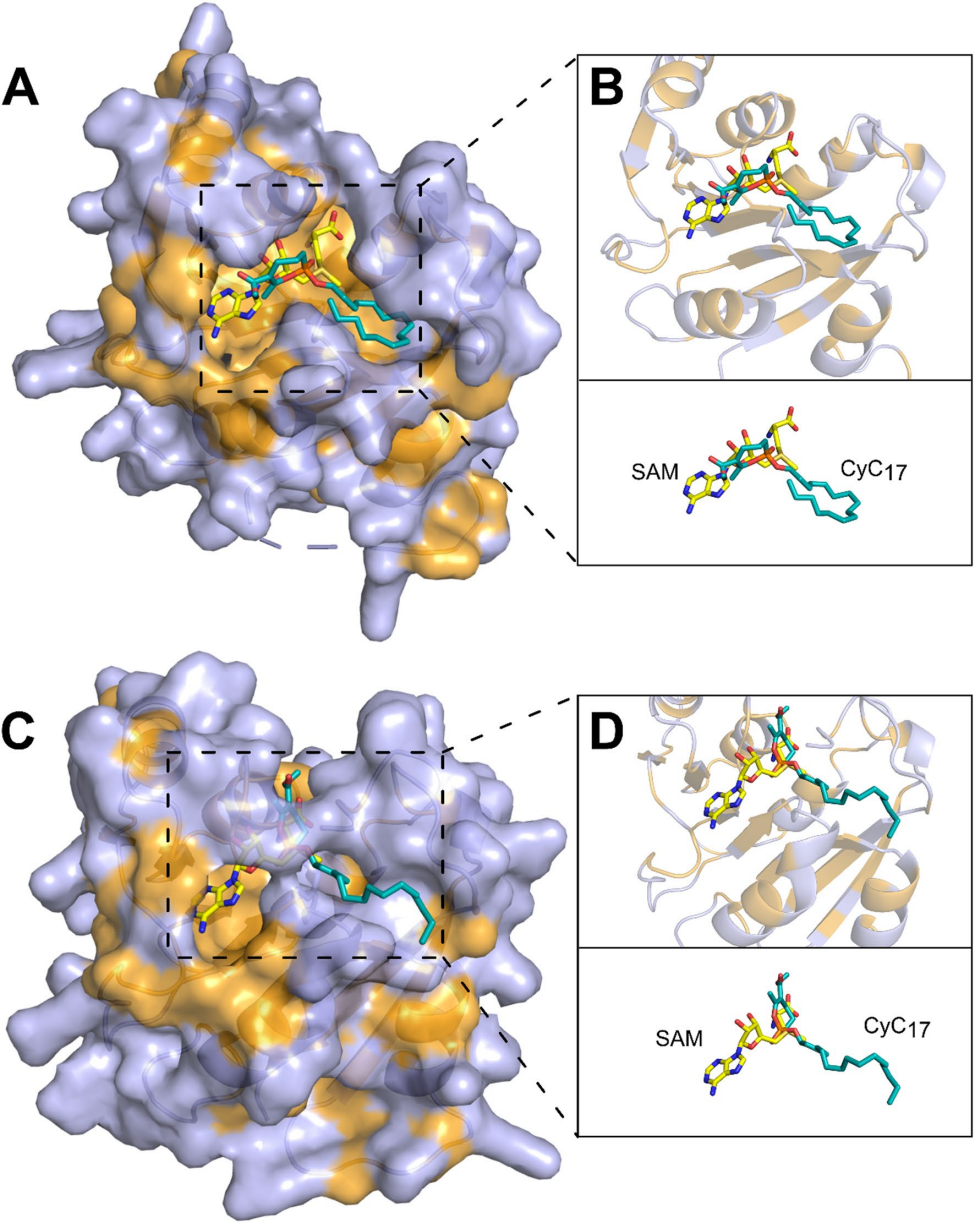
Molecular docking of CyC 17 in the active site of Erm(41). Model structures of Erm(41) generated using the Phyre2 web portal for protein modeling and based on **A** , **B** Erm(E) structure (PDB id: 6NVM) or C , D Erm(38) structure (PDB id: 7F8B). **B** , **D** Catalytic pocket view in presence of **CyC**_**17**_ and SAM co-factor. Hydrophobic residues (alanine, leucine, isoleucine, valine, tryptophan, tyrosine, phenylalanine, proline and methionine) are highlighted in orange. The inhibitor and SAM cofactor are in stick representation with the following atom color-code: oxygen red; phosphorus orange; carbon cyan for **CyC**_**17**_ and yellow for SAM. Structures were drawn with PyMOL Molecular Graphics System (version 1.4, Schrödinger, LLC)

To identify the potential binding site of the **CyC** into the protein, we generated three dimensional structural models of Erm(41) based on the *Saccharopolyspora erythraea* Erm(E) (PDB id: 6NVM) and on the *M. smegmatis* Erm(38) (PDB id: 7F8B) crystal structures, Erm(E) and Erm(38) share 28% and 30% sequence identity with Erm(41), respectively [51, 52]. Both models which are based on high-resolution 3D structures (1.75–2.25 Å), share sequence coverage of 92% with Erm(41), ensuring a proper orientation of the amino acid side chains (Fig. 2). Although these two models generated slightly different 3D structures (RSMD 1.12 Å), both unraveled an Y-shaped catalytic pocket able to accommodate the *S*-adenosyl-L-methionine (SAM) co-factor. SAM is the methyl donor for Erm(41) to methylate the 23 S rRNA at the adenine 2270 [53, 54]. The Erm(41) pocket comprises mostly hydrophobic and positively-charge residues at its entrance, facilitating the interaction with the negatively-charged rRNA, for the methyltransferase reaction.

The docking of **CyC**_**17**_ in the Erm(41) pocket generated from the two structural models revealed that this **CyC** would adopt a productive orientation that could prevent the positioning of SAM in active site (Fig. 2B and D). Taken together, these 3D models along with the biochemical data not only validate the ability of **CyC**_**17**_ to interact with Erm(41), but importantly, suggest that its location inside the catalytic pocket prevents the accommodation of SAM, rendering the enzyme inactive.

**Figure. 3:**
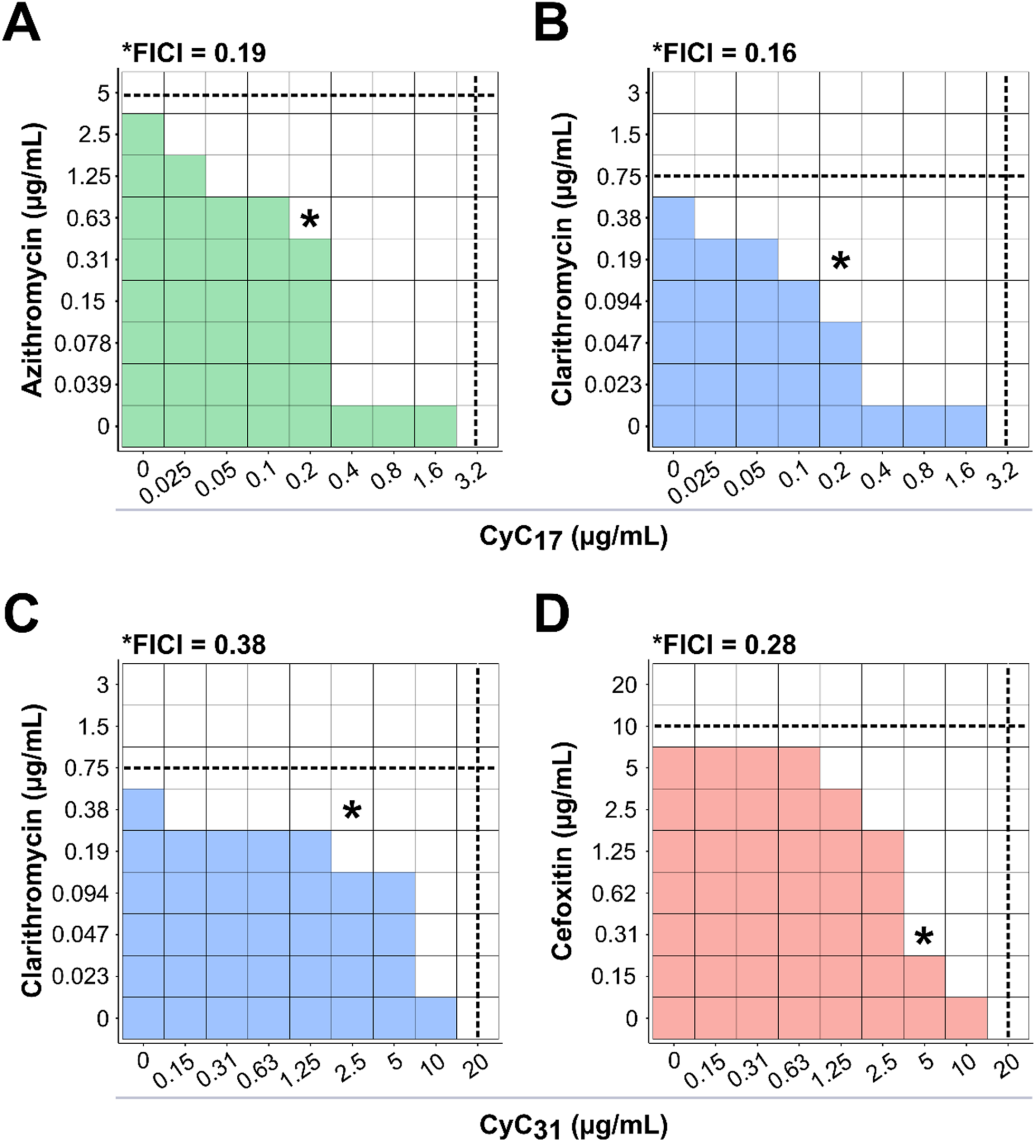
Synergistic activity of CyC-antibiotic combinations against *M. abscessus*. Schematic checkerboard representation of association between **A** azithromycin or **B** clarithromycin and **CyC**_**17**_ and between **C** clarithromycin or **D** cefoxitin and **CyC**_**31**_. The growth is represented by the colored boxes. Black dotted lines represent the respective MIC of the drug (horizontal) and the **CyC** (vertical). The calculated fractional inhibitory concentration index (FICI) of the best combination is indicated on top of each representation and also by a black star. The smallest FICI obtained for a combination is taken as the FICI of the association. Data are representative of three independent biological replicates

### Combined efficacity of the CyC with other drugs on macrolide-susceptible strains

We next investigated the in vitro interaction of the **CyC** in association with a panel of drugs used in clinical settings: CLR, AZM, AMK, IPM and CFX. The **CyC**_**17**_ and **CyC**_**31**_ which are the best inhibitors of extracellularly-growing *M. abscessus* [26] have been selected and tested with CLR and AZM using the checkerboard assay. The MIC of each compound tested alone was 5 µg/mL for AZM, 0.75 µg/mL for CLR, 3.2 µg/mL for **CyC**_**17**_ and 20 µg/mL for **CyC**_**31**_. The association of AZM with **CyC**_**17**_ showed a clear synergistic effect with a fractional inhibitory concentration index (FICI) of 0.19 (Fig. 3A). In this context, the best combination displayed MIC_AZM_ and MIC_CyC17_ values that were 8-fold (0.63 µg/mL) and 16-fold lower (0.2 µg/mL) than that of AZM and **CyC**_**17**_ alone, respectively. Also, the association of CLR with **CyC**_**17**_ and **CyC**_**31**_ showed a synergistic effect with a FICI of 0.16 and 0.38 respectively (Fig. 3B and C). In this context, the best combination displayed MIC_CLR_ and MIC_CyC17_ values that were 8-fold (0.094 µg/mL) and 32-fold lower (0.025 µg/mL) than that of CLR and **CyC**_**17**_ alone, respectively. About **CyC**_**31**_-CLR association, the MIC_CLR_ and MIC_CyC31_ values were 4-fold (0.19 µg/mL) and 128-fold lower (0.15 µg/mL) than that of CLR and **CyC**_**31**_ alone, respectively. Regarding the **CyC**_**31**_-AZM association, no effect could be measured on either compound for every combination, with calculated FICI between 0.63 and 2.5, the interaction was qualified as indifferent (Figure S1).

**Fig. 4:**
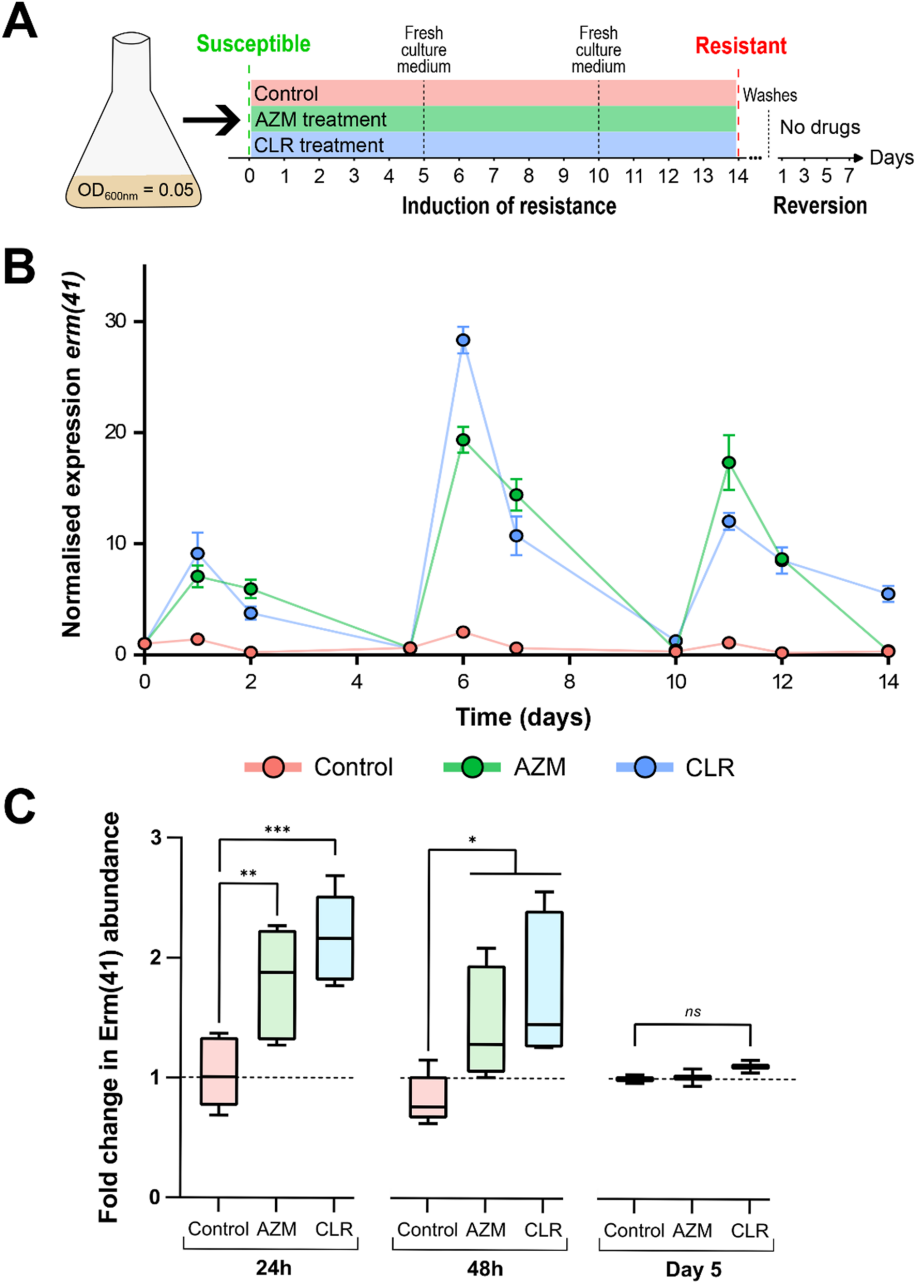
Induction of macrolide resistance. **A** Schematic protocol of induction of macrolide resistance and reversion in *M. abscessus* strains **B** Expression of erm(41) transcripts was quantified by RT-qPCR during the induction of macrolide resistance in *M. abscessus*. The amount of transcript in the untreated condition (control) at day 0 is taken as reference. Data represent the mean of normalized expression of two biological replicates. **C** Quantification of Erm(41) abundance by mass spectrometry. The graph shows the variations in fold change values for Erm(41) abundance after 24 h, 48 h and 5 days of induction of macrolide resistance in *M. abscessus* . In each case, the untreated control strain was taken as reference with a fold change of 1. Statistical analysis was done using a non-parametric Mann–Whitney test with Prism 8.0 (Graphpad Inc): * p value < 0.05; ** p value < 0.01; *** p value < 0.001

We next evaluated the in vitro activity of AMK, IPM or CFX in association with **CyC**_**17**_ or **CyC**_**31**_. The MIC values of AMK, IPM and CFX alone were 10 µg/mL, > 20 µg/mL and 10 µg/mL, respectively, in agreement with published values [55]. As observed above with AZM and CLR, the combination of CFX with **CyC**_**31**_ showed a synergistic effect, with a FICI of 0.28 (Fig. 3D), resulting in a 32-fold (0.31 µg/mL) and 4-fold (5 µg/mL) decrease in MIC values of CFX and **CyC**_**31**_ respectively. For the other combinations, including **CyC**_**17**_-AMK, **CyC**_**31**_-AMK, **CyC**_**17**_-IPM, **CyC**_**31**_-IPM and **CyC**_**17**_-CFX, the FICI values were between 0.51 and 2.0, considered as indifferent interactions (Figure S1). However, it should be noted that in the **CyC**_**31**_-AMK association, the presence of **CyC**_**31**_ allowed a 2‑fold decrease in MIC_AMK_ in 0.15–1.25 µg/mL interval and a 4‑fold decrease in MIC_AMK_ in 2.5–10 µg/mL interval.

Globally, these results suggest that the **CyC** exert a positive effect when associated with other antibiotics, notable with AZM, CLR and CFX with FICI ranging between 0.16 and 0.38. These drug combinations reduced by at least a 4-fold the MIC of the antibiotics.

**Fig. 5:**
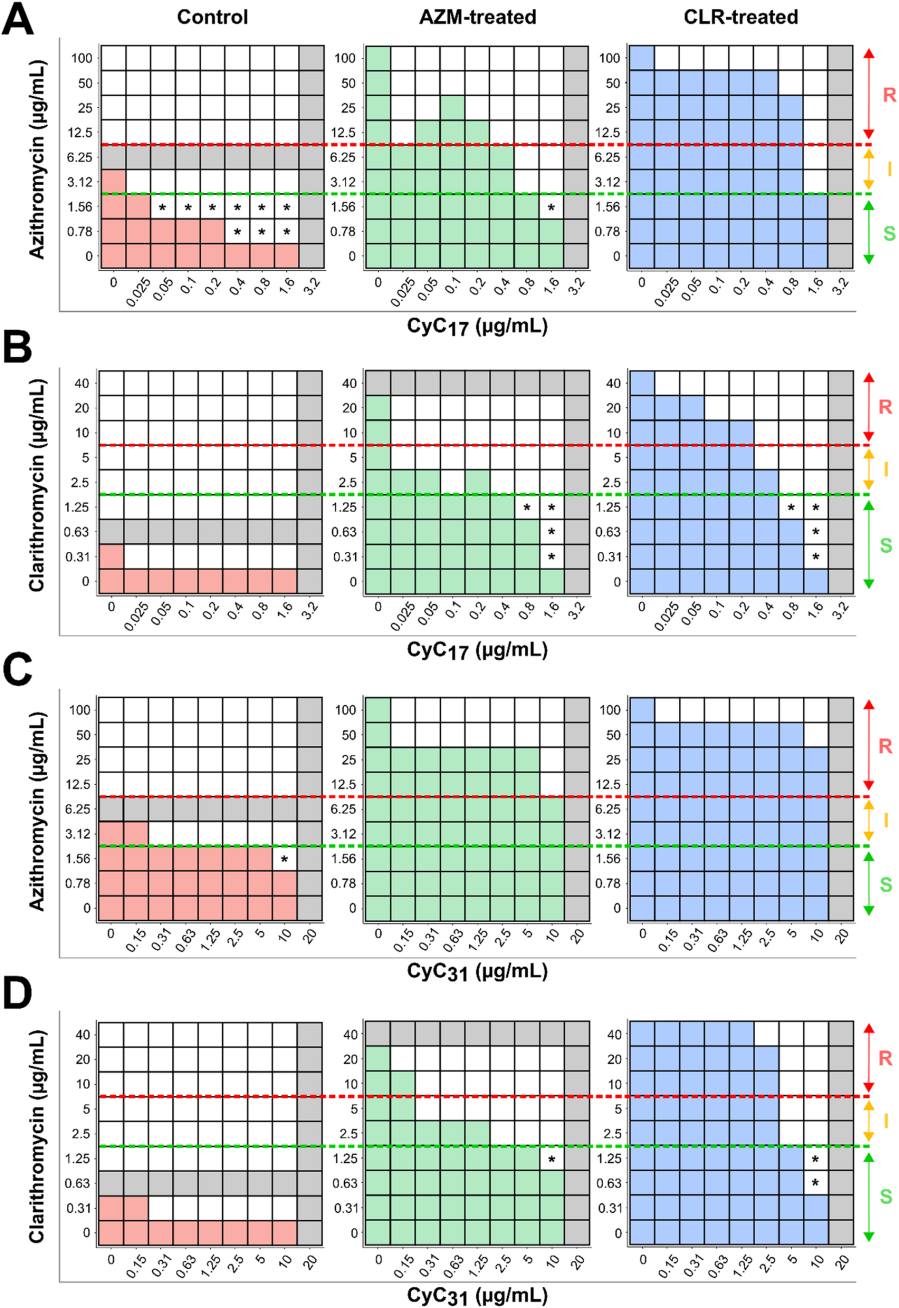
Activity of CyC-macrolides association on macrolide-resistant *M. abscessus* strains. Schematic checkerboard representation of association between **A** azithromycin or **B** clarithromycin and **CyC** **17** , and between **C** azithromycin or **D** clarithromycin and CyC **31** using WT (Control), AZM- and CLR-treated strains. Bacterial growth is represented by colored boxes, and the corresponding MIC values for macrolides and CyC are shown in gray. Based on CLSI guidelines, the green dotted lines represent the maximum macrolide concentration for which the strain is considered susceptible, and the red dotted lines correspond to the minimum concentration for which the strain is considered resistant. If bacterial growth is above the red line, the phenotype is considered resistant to macrolides, below the green line, the phenotype is considered susceptible, and between the two lines, the phenotype is intermediate. The new susceptible macrolides conditions have been indicated by a black star. S susceptible, I intermediate and R resistant

### Induction of macrolide resistance and*erm(41) *expression

The validation of Erm(41) as a target of the **CyC** inhibitors prompted us to evaluate their capacity to block the Erm(41)-mediated inducible macrolide resistance. To do so, *M. abscessus* was first pre-exposed to CLR or AZM for 14 days, as previously reported [13–17]. To avoid the generation of spontaneous resistant mutant in *rrl* gene (*MAB_r5052*), coding for rRNA 23 S which is the main target of macrolides, *M. abscessus* was incubated with sub-inhibitory macrolide concentrations corresponding to 0.1×MIC, i.e., 0.1 µg/mL and 0.5 µg/mL for CLR and AZM, respectively (Fig. 4A). The inducible macrolide resistance was followed by MIC measurements, Western blotting, global proteomic analysis and determination of the *erm(41)* transcriptional levels, as reported [13–15]. Determination of MIC was assessed after 5, 10 and 14 days of treatment (Table 1). As expected, the prolonged treatment with CLR or AZM increased both the MIC_CLR_ and MIC_AZM_ values up to 50times (≥ 40 µg/mL) and 17-times (> 100 µg/mL), respectively, after 14 days for both CLR-treated and AZM-treated strains. As noticed earlier [17], the MIC of AZM increased more rapidly than the one of CLR. These results confirm that pre-exposure of *M. abscessus* to sub-MIC concentrations of CLR or AZM induces macrolide resistance. Conversely, when performed on *M. massiliense* which lacks a functional Erm(41) protein [13], no resistance was observed (MIC_AZM_ = 1.03 ± 0.10 µg/mL; MIC_CLR_ = 1.45 ± 0.02 µg/mL) even after 14 days of treatment, which validates the macrolide induction protocol.

**Table 1:** Resistance levels to macrolides following exposure of *M. abscessus *to CLR or AZM

	MIC (µg/mL)									
Growth conditions	Before exposition		Day 5		Day 10		Day 14		Reversion	
	CLR	AZM	CLR	AZM	CLR	AZM	CLR	AZM	CLR	AZM
Control (Untreated)	0.9 ± 0.5	5.8 ± 1.6	0.8 ± 0	6.9 ± 1.4	0.3 ± 0.1	4.5 ± 1.5	0.6 ± 0.2	6.0 ± 2.0	ND	ND
CLR	0.9 ± 0.5	5.8 ± 1.6	3.1 ± 1.7	47 ± 17	9.4 ± 3.4	> 100	> 40	> 100	0.8 ± 0.4	4.1 ± 1.2
AZM	0.9 ± 0.5	5.8 ± 1.6	3.6 ± 2.1	63 ± 0	8.3 ± 3.2	> 100	40 ± 11	> 100	0.7 ± 0.2	3.6 ± 1.1

The MICs are given in µg/mL. For the reversion experiments, resistant mutants obtained after 14 days of induction were washed with fresh medium, grown without antibiotic for 7 day and susceptibility testing was performed to check the capacity of the strains to revert to a susceptible phenotype. The MIC presented in the table correspond to the mean ± SD of the MIC obtained from three independent biological replicates. *ND* not determined

In parallel, transcriptional expression of *erm(41)* was monitored during the 14 days of exposure to CLR or AZM. The *erm(41)* mRNA levels were quantified by reverse transcription PCR at day 1, 2, 5, 6, 7, 10, 11, 12 and 14, with drug renewal every 5 days **(**Fig. 4A**)**. As shown in Fig. 4B, subMIC macrolide concentrations induced *erm(41)* expression with a peek at 24 h, followed by a progressive decrease until day 5. Antibiotic renewal significantly increased the mRNA level of *erm(41)* for both macrolides although following a similar *erm(41)* gene expression kinetic (rapid induction at day 6 and progressive decrease up to day 10). These results demonstrate the importance to renew macrolides during the induction of resistance phase in order to maintain high *erm(41)* expression levels. Next, *M. abscessus* lysates from cultures exposed to CLR or AZM for 24 h and 48 h, respectively, were probed using Erm(41)-specific murine antibodies. However, we were unable to detect the protein in these samples despite several attempts (data not show). Thus, Erm(41) detection was done by analyzing the complete proteome of CLR- and AZM-treated *M. abscessus* cultures after 24 h, 48 h and 5 days of exposure. Statistical differential analysis with the non-treated *M. abscessus* proteome showed the presence of Erm(41) protein in the AZM- and CLR-treated *M. abscessus* proteome at 24 h and 48 h but not at 5 days of treatment to macrolides (Fig. 4C). These results confirm the RT-qPCR results, but also show a weak overexpression of the protein with a fold-change of 1.7–2.1 at 24 h and 1.3–1.6 at 48 h compared to the proteome of non-treated cultures. The presence of the Erm(41) at 24 and 48 h, together with the low level of *erm(41)* transcripts and its disappearance at day 5, suggests that this protein may be produced in low scale and is probably very unstable.

To evaluate whether inducible macrolide resistance mechanism is reversible, macrolide-resistant strains were washed with fresh 7H9-S^OADC^ medium and grown in culture medium without antibiotic for 7 days [17]. Then, susceptibility testing was performed to check the capacity of the strains to revert to a susceptible phenotype (Table 1, Reversion condition). Whatever the macrolide used for the induction of resistance, MIC of AZM and CLR measured after 7 days of culture medium without any antibiotic were identical to those before macrolide induction, confirming that this resistance phenomenon is reversible. Moreover, no mutation was found in *rrl* is AZM- or CLR-treated strains (data not show), thus excluding the emergence of a genetically-acquired resistance mechanism.

In contrast, we generated a spontaneous macrolide-resistant mutant strain by using a high concentration of macrolide (10× MIC), displaying high level of resistance to CLR (MIC > 40 µg/mL) and AZM (MIC > 100 µg/mL). This mutant carries an adenine-to-guanine replacement at position 2271 in *rrl* avoiding the macrolide to interact with the 23 S rRNA (Figure S2), as already reported in *M. abscessus* clinical isolates [56, 57].

### CyC-macrolide association on macrolide-resistant strains

The ability of the **CyC** to interact in vitro with purified Erm(41) protein suggest that these inhibitors could antagonize the Erm(41) activity and, consequently, restore susceptibility to macrolides. To test this hypothesis, we evaluated in vitro the association of AZM and CLR with **CyC**_**17**_ or **CyC**_**31**_ using the checkerboard assay with reversible resistant strains generated as described below (Fig. 5, Table S1 and S2). For susceptibility breakpoints, we followed the Clinical and Laboratory Standards Institute (CLSI) guidelines [58].

The control strain, corresponding to the untreated condition, showed already an intermediate AZM sensitivity profile (MIC_AZM_ = 6 µg/mL). The association of **CyC**_**17**_ with AZM resulted in a 3- and 7-fold decrease in MIC_AZM_ in the 0.05–0.2 µg/mL (MIC_AZM_ = 2 µg/mL) and in the 0.4–1.6 µg/mL (MIC_AZM_ = 0.8 µg/mL) **CyC**_**17**_ concentration range, respectively. Similarly, a 2-fold reduction in MIC_AZM_ (MIC_AZM_ = 3 µg/mL) was reached in presence of **CyC**_**31**_ in the 0.31–5 µg/mL concentration range, the strain becoming fully susceptible from 10 µg/mL of **CyC**_**31**_. Although the addition of **CyC**_**17**_ and **CyC**_**31**_ with CLR did not modify the CLR susceptibility profile of the control strain, their presence led to a 2-fold reduction in CMI_CLR_.

As reported above resistant strains displayed a MIC_AZM_ >100, MIC_CLR_ >40 µg/mL, MIC_**CyC17**_ = 3.2 µg/mL and MIC_**CyC31**_ = 20 µg/mL, respectively.

For the CLR-treated strain, two combinations were able to restore susceptibility to macrolides. The association of CLR with **CyC**_**17**_ leads to a gradual decrease of MIC_CLR_ until an intermediate susceptibility profile at 0.4 µg/mL, corresponding to **CyC**_**17**_’s MIC_/8_. The strain become fully susceptible at CyC_17_’s MIC_/4_ concentration with a fold change reduction of 40 (MIC_CLR_ = 1 µg/mL), and even reached a fold change in its MIC_CLR_ up to 133 (MIC_CLR_ = 0.3) at **CyC**_**17**_’s MIC_/2_. Similarly, 5 µg/mL (i.e., MIC_/4_) **CyC**_**31**_ allowed to dramatically decrease the MIC_CLR_ (3 µg/mL) by 13-fold. When using 10 µg/mL (i.e., **CyC**_**31**_’s MIC_/2_) the strain became fully susceptible to CLR with a fold change of 66 in its MIC_CLR_.

In the case of AZM-treated strains, three combinations were able to restore susceptibility to macrolides. The AZM association with 0.8 µg/mL (MIC_/4_) of **CyC**_**17**_ allowed to reach a MIC_AZM_ value of 3 µg/mL (33-fold decrease), which is considered as ‘intermediate’. In the presence of 1.6 µg/mL (MIC_/2_) of **CyC**_**17**_ a 50-fold decrease in MIC_AZM_ was reached (MIC_AZM_=2 µg/mL) and the strain was considered susceptible. With the **CyC**_**31**_, regardless the concentration used, the AZM-treated strain remained resistant, even if a 7-fold decrease in MIC_AZM_ was obtained at a **CyC**_**31**_’s MIC_/2_. Regarding CLR susceptibility to this AZM-treated strain, the most efficient associations was the combination of CLR with **CyC**_**17**_ or **CyC**_**31**_. The addition of low concentration of **CyC**_**17**_ corresponding to MIC_/64_ decreased drastically MIC_CLR_ until an intermediate level. The use of **CyC**_**17**_’s MIC_/4_ concentration (0.8 µg/mL) fully restored CLR susceptibility. Similarly, the addition of **CyC**_**31**_ together with CLR resulted in an 8-fold decrease in MIC_CLR_ for CyC_31_ concentrations ranging from 0.31 to 1.25 µg/mL. Finally, 10 µg/mL of **CyC**_**31**_ (MIC_/2_) rendered the strain susceptible to CLR.

On the other hand, when testing the association of AZM and CLR with the **CyC**_**17**_ on *M. massiliense*, no effect was observed with respect to the MICs of AZM (0.625–1.25 µg/mL), CLR (0.093–0.188 µg/mL) or the **CyC**_**17**_ (3.2 µg/mL). Aligning with these results, the activity of **CyC**_**17**_ and **CyC**_**31**_ with macrolides against the macrolide-resistant spontaneous mutant carrying an irreversible point mutation in *rrl* gene did not influence the susceptibility profile to CLR or AZM (Table S3). Overall, the results based on *M. massiliense* and the *rrl* mutant suggest that the potentiating effect of the **CyC** for macrolides is only effective in the case of Erm(41)-dependent inducible resistance.

## Discussion

*M. abscessus* is considered as the main pathogenic RGM in humans [59], responsible for a broad spectrum of infections ranging from mucocutaneous infections in immunocompetent individuals to severe pulmonary infections in patients with CF or chronic obstructive pulmonary disease [2, 3]. The natural resistance of *M. abscessus* to most conventional antibiotics, the poor clinical outcome and the lengthy regimens render treatments particularly difficult. In clinical practice, drug combinations are given in efforts to prevent the development of drug resistance during therapy and to optimize the efficacy of the treatments. In this context, the discovery of a new family of the multitargeted **CyC** inhibitors, acting specifically on mycobacteria with no toxicity toward mammalian cells, adds new hope for subsequent therapeutic developments [26–28]. Among the targets impacted by the **CyC**, we identified the Erm(41) a methyltransferase involved in the resistance induced by macrolides, which are the pillar of anti-*M. abscessus* therapy and whose inactivation often leads to a therapeutic end [1, 20]. Here, we investigated the interaction between the **CyC** and Erm(41) to bypass inducible macrolide resistance, thus opening the door to new therapeutic interventions.

Foremost, we showed that Erm(41) interacts with several **CyC**, including **CyC**_**17**_ and **CyC**_**31**_ which exhibit *M. abscessus* growth in vitro growth; and **CyC**_**8α**_ and **CyC**_**8β**_, primarily acting against *M. abscessus* residing in the macrophage [26, 29]. Biochemical studies involving the TAMRA-FP probe that binds to serine hydrolases along with mass spectrometry and in silico molecular docking, suggest that the **CyC** analogues are covalently bound to the active site of Erm(41), thereby preventing the accommodation of SAM into Erm(41) the catalytic site, resulting in enzymatic blockage. The future high-resolution structure of Erm(41) and detailed description of the catalytic mechanism of the enzyme may lead to the development of other chemical entities capable to inhibit its enzymatic activity. Alternatively, the development of other classes of Erm(41)specific inhibitors or SAM derivatives may provide means to preserve high susceptibility levels of *M. abscessus* isolates to macrolides [60, 61].

We investigated the synergistic potential of the **CyC** in combination with multiple antimicrobials active against *M. abscessus*. Several combinations were tested in vitro with **CyC**_**17**_ and **CyC**_**31**_, emphasizing optimal synergistic interactions between **CyC**_**17**_-AZM, **CyC**_**17**_-CLR and **CyC**_**31**_**-**CLR. Our data also highlighted the potent association between **CyC**_**31**_ and CFX, a drug largely used for the treatment against *M. abscessus* infections. Overall, this study confirms that the **CyC** can be used in association with other clinically relevant drugs against this species, displaying a clear improvement regarding the existing treatments, at least in vitro. Pre-clinical studies are now warranted to study the impact and efficacy of these treatment combinations in animal models.

Importantly, the **CyCs** are able to counteract macrolide resistance mediated by Erm(41) or remain active on spontaneous resistant mutants carrying a mutation in the *rrl* gene. This restoration of macrolide susceptibility is of prime interest and offers new prospects for the development of drug regimens.

In this study, we demonstrated that the Erm(41) protein is also able to interact with the **CyC**_**8(α,β)**_ which are only active ex vivo inside infected macrophages [26]. These results may suggest that these **CyC**_**8(α,β)**_ might also be able to restore macrolide susceptibility in resistant strains generated by a treatment to macrolides. To confirm this hypothesis, it will be necessary to test the association of **CyC**_**8(α,β)**_ with AZM or CLR against macrophages infected with *M. abscessus* strain whose resistance has previously been induced.

Finally, our data confirmed the efficacy of the **CyC** to restore the CLR or AZM susceptibility on macrolide-resistant *M. abscessus* strain following Erm(41) induction. A major asset of the **CyC** relies on the fact that these molecules can target multiple enzymes, thus preventing the emergence of spontaneous resistant mutants. The work presented here describes a novel and unique aspect of this family of inhibitors by lowering/suppressing a mechanism that leads to drug in inactivation. To the best of our knowledge, **CyC** are the first inhibitors of the Erm(41) methyltransferase.

In addition, this work revealed that the synergy activity of the **CyC** in association with CFX should also be taken into consideration. Further experiments are, however, needed to better understand the relationship between the CFX and **CyC** compounds.

The recently studied CLR-Rifabutin combination showed also a synergistic effect able to block Erm(41) induction and so increased the efficiency of macrolide [24]. Thereby, blocking the main actor responsible for the resistance to macrolide by molecules unable to generate spontaneous mutants deserves more attention.

## Conclusion

These results provide the opportunity to revisit the therapeutic regimen for combating *M. abscessus* pulmonary infections in CF patients, and particularly *erm(41)*-positive strains. Long-term prospects should help to expand our therapeutic arsenal in the fight against a particularly antibioticresistant mycobacterial species.

## Supplementary Information

Additional file 1.

## Data Availability

All data generated and analyzed during this study are included in this article.

## Abbreviations

ABPP: Activity-based protein profiling
AMK: Amikacin
AZM: Azythromycin
CF: Cystic fibrosis
CLR: Clarithromycin
CFX: Cefoxitin
CyC: Cyclipostins and Cyclophostin analogues
IPM: Imipenem
FIC: Fractional inhibitory concentration
FICI: Fractional inhibitory concentration index
MIC: Minimal inhibitory concentration
RGM: Rapid-growing mycobacteria

## References

[1] Griffith DE, Aksamit T, Brown-Elliott BA, Catanzaro A, Daley C, Gordin F, Holland SM, Horsburgh R, Huitt G, Iademarco MF, Iseman M, Olivier K, Ruoss S, von Reyn CF, Wallace RJ Jr, Winthrop K. An official ATS/IDSA statement: diagnosis, treatment, and prevention of nontuberculous mycobacterial diseases. Am J Respir Crit Care Med. 2007;175(4):367–416. 17277290 10.1164/rccm.200604-571ST

[2] Cowman S, van Ingen J, Griffith DE, Loebinger MR. Non-tuberculous mycobacterial pulmonary disease. Eur Respir J. 2019. 10.1183/13993003.00250-2019. 31221809 10.1183/13993003.00250-2019

[3] Martiniano SL, Nick JA, Daley CL. Nontuberculous mycobacterial infections in cystic fibrosis. Clin Chest Med. 2022;43(4):697–716. 36344075 10.1016/j.ccm.2022.06.010

[4] Wallace RJ Jr., Brown-Elliott BA, Crist CJ, Mann L, Wilson RW. Comparison of the in vitro activity of the glycylcycline tigecycline (formerly GAR-936) with those of tetracycline, minocycline, and doxycycline against isolates of nontuberculous mycobacteria. Antimicrob Agents Chemother. 2002;46(10):3164–7. 12234839 10.1128/AAC.46.10.3164-3167.2002 PMC128779

[5] Nessar R, Cambau E, Reyrat JM, Murray A, Gicquel B. Mycobacterium abscessus: a new antibiotic nightmare. J Antimicrob Chemother. 2012;67(4):810–8. 22290346 10.1093/jac/dkr578

[6] Maurer FP, Bruderer VL, Castelberg C, Ritter C, Scherbakov D, Bloemberg GV, Bottger EC. Aminoglycoside-modifying enzymes determine the innate susceptibility to aminoglycoside antibiotics in rapidly growing mycobacteria. J Antimicrob Chemother. 2015;70(5):1412–9. 25604746 10.1093/jac/dku550

[7] Lefebvre AL, Le Moigne V, Bernut A, Veckerle C, Compain F, Herrmann JL, Kremer L, Arthur M, Mainardi JL. Inhibition of the beta-lactamase bla(Mab) by avibactam improves the in vitro and in vivo efficacy of imipenem against *Mycobacterium abscessus*. Antimicrob Agents Chemother. 2017. 10.1128/aac.02440-16. 28096155 10.1128/AAC.02440-16 PMC5365697

[8] Rominski A, Roditscheff A, Selchow P, Bottger EC, Sander P. Intrinsic rifamycin resistance of *Mycobacterium abscessus* is mediated by ADP-ribosyltransferase MAB_0591. J Antimicrob Chemother. 2017;72(2):376–84. 27999011 10.1093/jac/dkw466

[9] Brown-Elliott BA, Vasireddy S, Vasireddy R, Iakhiaeva E, Howard ST, Nash K, Parodi N, Strong A, Gee M, Smith T, Wallace RJ Jr. Utility of sequencing the erm(41) gene in isolates of *Mycobacterium abscessus* subsp. abscessus with low and intermediate clarithromycin MICs. J Clin Microbiol. 2015;53(4):1211–5. 25653399 10.1128/JCM.02950-14 PMC4365201

[10] Daley CL, Iaccarino JM, Lange C, Cambau E, Wallace RJ, Andrejak C, Bottger EC, Brozek J, Griffith DE, Guglielmetti L, Huitt GA, Knight SL, Leitman P, Marras TK, Olivier KN, Santin M, Stout JE, Tortoli E, van Ingen J, Wagner D, Winthrop KL. Treatment of nontuberculous mycobacterial pulmonary disease: an official ATS/ERS/ESCMID/IDSA clinical practice guideline. Clin Infect Dis. 2020;71(4):905–13. 32797222 10.1093/cid/ciaa1125 PMC7768745

[11] Floto RA, Olivier KN, Saiman L, Daley CL, Herrmann JL, Nick JA, Noone PG, Bilton D, Corris P, Gibson RL, Hempstead SE, Koetz K, Sabadosa KA, Sermet-Gaudelus I, Smyth AR, van Ingen J, Wallace RJ, Winthrop KL, Marshall BC, Haworth CS. US Cystic Fibrosis Foundation and European Cystic Fibrosis Society consensus recommendations for the management of non-tuberculous mycobacteria in individuals with cystic fibrosis. Thorax. 2016;71(Suppl 1):i1-22. 26666259 10.1136/thoraxjnl-2015-207360 PMC4717371

[12] Choi H, Kim SY, Kim DH, Huh HJ, Ki CS, Lee NY, Lee SH, Shin S, Shin SJ, Daley CL, Koh WJ. Clinical characteristics and treatment outcomes of patients with acquired macrolide-resistant *Mycobacterium abscessus* lung disease. Antimicrob Agents Chemother. 2017;61(10):10–1128. 10.1128/AAC.01146-17 PMC5610486 28739795

[13] Nash KA, Brown-Elliott BA, Wallace RJ Jr. A novel gene, erm(41), confers inducible macrolide resistance to clinical isolates of *Mycobacterium abscessus* but is absent from *Mycobacterium chelonae*. Antimicrob Agents Chemother. 2009;53(4):1367–76. 19171799 10.1128/AAC.01275-08 PMC2663066

[14] Choi GE, Shin SJ, Won CJ, Min KN, Oh T, Hahn MY, Lee K, Lee SH, Daley CL, Kim S, Jeong BH, Jeon K, Koh WJ. Macrolide treatment for *Mycobacterium abscessus* and *Mycobacterium massiliense* infection and inducible resistance. Am J Respir Crit Care Med. 2012;186(9):917–25. 22878281 10.1164/rccm.201111-2005OC

[15] Maurer FP, Castelberg C, Quiblier C, Bottger EC, Somoskovi A. Erm(41)-dependent inducible resistance to azithromycin and clarithromycin in clinical isolates of *Mycobacterium abscessus*. J Antimicrob Chemother. 2014;69(6):1559–63. 24500188 10.1093/jac/dku007

[16] Schildkraut JA, Pennings LJ, Ruth MM, de Brouwer AP, Wertheim HF, Hoefsloot W, de Jong A, van Ingen J. The differential effect of clarithromycin and azithromycin on induction of macrolide resistance in *Mycobacterium abscessus*. Future Microbiol. 2019;14:749–55. 31271060 10.2217/fmb-2018-0310

[17] Richard M, Gutierrez AV, Kremer L. Dissecting erm(41)-mediated macrolide-inducible resistance in *Mycobacterium abscessus*. Antimicrob Agents Chemother. 2020;64(2):10–128. 10.1128/AAC.01879-19 PMC6985735 31791943

[18] Kim HY, Kim BJ, Kook Y, Yun YJ, Shin JH, Kook YH. *Mycobacterium massiliense* is differentiated from *Mycobacterium abscessus* and *Mycobacterium bolletii* by erythromycin ribosome methyltransferase gene (erm) and clarithromycin susceptibility patterns. Microbiol Immunol. 2010;54(6):347–53. 20536733 10.1111/j.1348-0421.2010.00221.x

[19] Harada T, Akiyama Y, Kurashima A, Nagai H, Tsuyuguchi K, Fujii T, Yano S, Shigeto E, Kuraoka T, Kajiki A, Kobashi Y, Kokubu F, Sato A, Yoshida S, Iwamoto T, Saito H. Clinical and microbiological differences between *Mycobacterium abscessus* and *Mycobacterium massiliense* lung diseases. J Clin Microbiol. 2012;50(11):3556–61. 22915613 10.1128/JCM.01175-12 PMC3486228

[20] Roux AL, Catherinot E, Soismier N, Heym B, Bellis G, Lemonnier L, Chiron R, Fauroux B, Le Bourgeois M, Munck A, Pin I, Sermet I, Gutierrez C, Veziris N, Jarlier V, Cambau E, Herrmann JL, Guillemot D, Gaillard JL. Comparing *Mycobacterium massiliense* and *Mycobacterium abscessus* lung infections in cystic fibrosis patients. J Cyst Fibros. 2015;14(1):63–9. 25085077 10.1016/j.jcf.2014.07.004

[21] Blondiaux N, Moune M, Desroses M, Frita R, Flipo M, Mathys V, Soetaert K, Kiass M, Delorme V, Djaout K, Trebosc V, Kemmer C, Wintjens R, Wohlkonig A, Antoine R, Huot L, Hot D, Coscolla M, Feldmann J, Gagneux S, Locht C, Brodin P, Gitzinger M, Deprez B, Willand N, Baulard AR. Reversion of antibiotic resistance in *Mycobacterium tuberculosis* by spiroisoxazoline SMARt-420. Science. 2017;355(6330):1206–11. 28302858 10.1126/science.aag1006

[22] Laws M, Shaaban A, Rahman KM. Antibiotic resistance breakers: current approaches and future directions. FEMS Microbiol Rev. 2019;43(5):490–516. 31150547 10.1093/femsre/fuz014 PMC6736374

[23] Dubee V, Bernut A, Cortes M, Lesne T, Dorchene D, Lefebvre AL, Hugonnet JE, Gutmann L, Mainardi JL, Herrmann JL, Gaillard JL, Kremer L, Arthur M. Beta-lactamase inhibition by avibactam in *Mycobacterium abscessus*. J Antimicrob Chemother. 2015;70(4):1051–8. 25525201 10.1093/jac/dku510

[24] Aziz DB, Go ML, Dick T. Rifabutin suppresses inducible clarithromycin resistance in *Mycobacterium abscessus* by blocking induction of whiB7 and erm41. Antibiot (Basel). 2020;9(2):72. 10.3390/antibiotics9020072 PMC7168051 32050554

[25] Cavalier JF, Spilling CD, Durand T, Camoin L, Canaan S. Lipolytic enzymes inhibitors: A new way for antibacterial drugs discovery. Eur J Med Chem. 2021;209:112908. 33071055 10.1016/j.ejmech.2020.112908

[26] Madani A, Ridenour JN, Martin BP, Paudel RR, Abdul Basir A, Le Moigne V, Herrmann JL, Audebert S, Camoin L, Kremer L, Spilling CD, Cavalier JF. Cyclipostins and cyclophostin analogues as multitarget inhibitors that impair growth of *Mycobacterium abscessus*. ACS Infect Dis. 2019;5(9):1597–608. 31299146 10.1021/acsinfecdis.9b00172

[27] Nguyen PC, Delorme V, Benarouche A, Martin BP, Paudel R, Gnawali GR, Madani A, Puppo R, Landry V, Kremer L, Brodin P, Spilling CD, Cavalier JF, Canaan S. Cyclipostins and Cyclophostin analogs as promising compounds in the fight against tuberculosis. Sci Rep. 2017;7(1):11751. 28924204 10.1038/s41598-017-11843-4 PMC5603573

[28] Nguyen PC, Madani A, Santucci P, Martin BP, Paudel RR, Delattre S, Herrmann JL, Spilling CD, Kremer L, Canaan S, Cavalier JF. Cyclophostin and cyclipostins analogues, new promising molecules to treat mycobacterial-related diseases. Int J Antimicrob Agents. 2018;51(4):651–4. 29241819 10.1016/j.ijantimicag.2017.12.001

[29] Sarrazin M, Martin BP, Avellan R, Gnawali GR, Poncin I, Le Guenno H, Spilling CD, Cavalier JF, Canaan S. Synthesis and biological characterization of fluorescent cyclipostins and cyclophostin analogues: new insights for the diagnosis of mycobacterial-related diseases. ACS Infect Dis. 2022;8(12):2564–78. 36379042 10.1021/acsinfecdis.2c00448

[30] Barelier S, Avellan R, Gnawali GR, Fourquet P, Roig-Zamboni V, Poncin I, Point V, Bourne Y, Audebert S, Camoin L, Spilling CD, Canaan S, Cavalier JF, Sulzenbacher G. Direct capture, inhibition and crystal structure of HsaD (Rv3569c) from *M. tuberculosis*. Febs J. 2023;290(6):1563–82. 36197115 10.1111/febs.16645

[31] Nguyen PC, Nguyen VS, Martin BP, Fourquet P, Camoin L, Spilling CD, Cavalier JF, Cambillau C, Canaan S. Biochemical and structural characterization of TesA, a major thioesterase required for outer-envelope lipid biosynthesis in *Mycobacterium tuberculosis*. J Mol Biol. 2018;430(24):5120–36. 30292819 10.1016/j.jmb.2018.09.017

[32] Point V, Malla RK, Diomande S, Martin BP, Delorme V, Carriere F, Canaan S, Rath NP, Spilling CD, Cavalier JF. Synthesis and kinetic evaluation of cyclophostin and cyclipostins phosphonate analogs as selective and potent inhibitors of microbial lipases. J Med Chem. 2012;55(22):10204–19. 23095026 10.1021/jm301216x PMC3518039

[33] Viljoen A, Richard M, Nguyen PC, Fourquet P, Camoin L, Paudal RR, Gnawali GR, Spilling CD, Cavalier JF, Canaan S, Blaise M. Cyclipostins and cyclophostin analogs inhibit the antigen 85 C from *Mycobacterium tuberculosis* both in vitro and in vivo. J Biol Chem. 2018;293(8):2755–69. 29301937 10.1074/jbc.RA117.000760 PMC5827452

[34] Dutta S, Malla RK, Bandyopadhyay S, Spilling CD, Dupureur CM. Synthesis and kinetic analysis of some phosphonate analogs of cyclophostin as inhibitors of human acetylcholinesterase. Bioorg Med Chem. 2010;18(6):2265–74. 20189400 10.1016/j.bmc.2010.01.063 PMC2841358

[35] Spilling CD. The chemistry and biology of cyclophostin, the cyclipostins and related compounds. Molecules. 2019;24(14):2579. 31315184 10.3390/molecules24142579 PMC6681047

[36] Martin BP, Vasilieva E, Dupureur CM, Spilling CD. Synthesis and comparison of the biological activity of monocyclic phosphonate, difluorophosphonate and phosphate analogs of the natural AChE inhibitor cyclophostin. Bioorg Med Chem. 2015;23(24):7529–34. 26585276 10.1016/j.bmc.2015.10.044

[37] Noens EE, Williams C, Anandhakrishnan M, Poulsen C, Ehebauer MT, Wilmanns M. Improved mycobacterial protein production using a *Mycobacterium smegmatis* groEL1DeltaC expression strain. BMC Biotechnol. 2011;11:27. 21439037 10.1186/1472-6750-11-27 PMC3076238

[38] Ripoll F, Pasek S, Schenowitz C, Dossat C, Barbe V, Rottman M, Macheras E, Heym B, Herrmann JL, Daffe M, Brosch R, Risler JL, Gaillard JL. Non mycobacterial virulence genes in the genome of the emerging pathogen *Mycobacterium abscessus*. PLoS One. 2009;4(6):e5660. 19543527 10.1371/journal.pone.0005660 PMC2694998

[39] Adekambi T, Reynaud-Gaubert M, Greub G, Gevaudan MJ, La Scola B, Raoult D, Drancourt M. Amoebal coculture of *Mycobacterium massiliense* sp. nov. from the sputum of a patient with hemoptoic pneumonia. J Clin Microbiol. 2004;42(12):5493–501. 15583272 10.1128/JCM.42.12.5493-5501.2004 PMC535245

[40] Bakala N, Schue JC, Carriere M, Geerlof F, Canaan S. Evidence for the cytotoxic effects of *Mycobacterium tuberculosis* phospholipase C towards macrophages. Biochim Biophys Acta. 2010;1801(12):1305–13. 20736081 10.1016/j.bbalip.2010.08.007

[41] Kelley LA, Mezulis S, Yates CM, Wass MN, Sternberg MJ. The Phyre2 web portal for protein modeling, prediction and analysis. Nat Protoc. 2015;10(6):845–58. 25950237 10.1038/nprot.2015.053 PMC5298202

[42] Bianco G, Forli S, Goodsell DS, Olson AJ. Covalent docking using autodock: two-point attractor and flexible side chain methods. Protein Sci. 2016;25(1):295–301. 26103917 10.1002/pro.2733 PMC4815316

[43] Trott O, Olson AJ. AutoDock Vina: improving the speed and accuracy of docking with a new scoring function, efficient optimization, and multithreading. J Comput Chem. 2010;31(2):455–61. 19499576 10.1002/jcc.21334 PMC3041641

[44] Seeliger D, de Groot BL. Ligand docking and binding site analysis with PyMOL and Autodock/Vina. J Comput Aided Mol Des. 2010;24(5):417–22. 20401516 10.1007/s10822-010-9352-6 PMC2881210

[45] Demichev V, Messner CB, Vernardis SI, Lilley KS, Ralser M. DIA-NN: neural networks and interference correction enable deep proteome coverage in high throughput. Nat Methods. 2020;17(1):41–4. 31768060 10.1038/s41592-019-0638-x PMC6949130

[46] Gerault MA, Camoin L, Granjeaud S. DIAgui: a Shiny application to process the output from DIA-NN. Bioinform Adv. 2024;4(1):vbae001. 38249340 10.1093/bioadv/vbae001 PMC10799745

[47] Le Run E, Arthur M, Mainardi JL. In vitro and intracellular activity of imipenem combined with rifabutin and avibactam against *Mycobacterium abscessus*. Antimicrob Agents Chemother. 2018;62(8):10–128. 10.1128/AAC.00623-18 PMC6105861 29866869

[48] Odds FC. Synergy, antagonism, and what the chequerboard puts between them. J Antimicrob Chemother. 2003;52(1):1. 12805255 10.1093/jac/dkg301

[49] Santucci P, Point V, Poncin I, Guy A, Crauste C, Serveau-Avesque C, Galano JM, Spilling CD, Cavalier JF, Canaan S. LipG a bifunctional phospholipase/thioesterase involved in mycobacterial envelope remodeling. Biosci Rep. 2018;38(6):BSR20181953. 30487163 10.1042/BSR20181953 PMC6435540

[50] Liu Y, Patricelli MP, Cravatt BF. Activity-based protein profiling: the serine hydrolases. Proc Natl Acad Sci USA. 1999;96(26):14694–9. 10611275 10.1073/pnas.96.26.14694 PMC24710

[51] Stsiapanava A, Selmer M. Crystal structure of ErmE – 23S rRNA methyltransferase in macrolide resistance. Sci Rep. 2019;9(1):14607. 31601908 10.1038/s41598-019-51174-0 PMC6787224

[52] Goh BC, Xiang X, Lescar J, Dedon PC. Crystal structure and functional analysis of mycobacterial erythromycin resistance methyltransferase Erm38 reveals its RNA-binding site. J Biol Chem. 2022;298(2):101571. 35007529 10.1016/j.jbc.2022.101571 PMC8844858

[53] Skinner R, Cundliffe E, Schmidt FJ. Site of action of a ribosomal RNA methylase responsible for resistance to erythromycin and other antibiotics. J Biol Chem. 1983;258(20):12702–6. 6195156

[54] Malone T, Blumenthal RM, Cheng X. Structure-guided analysis reveals nine sequence motifs conserved among DNA amino-methyltransferases, and suggests a catalytic mechanism for these enzymes. J Mol Biol. 1995;253(4):618–32. 7473738 10.1006/jmbi.1995.0577

[55] Singh S, Bouzinbi N, Chaturvedi V, Godreuil S, Kremer L. In vitro evaluation of a new drug combination against clinical isolates belonging to the *Mycobacterium abscessus* complex. Clin Microbiol Infect. 2014;20(12):O1124-1127. 25185732 10.1111/1469-0691.12780

[56] Wallace RJ Jr, Meier A, Brown BA, Zhang Y, Sander P, Onyi GO, Bottger EC. Genetic basis for clarithromycin resistance among isolates of *Mycobacterium chelonae* and *Mycobacterium abscessus*. Antimicrob Agents Chemother. 1996;40(7):1676–81. 8807061 10.1128/aac.40.7.1676 PMC163394

[57] Lipworth S, Hough N, Leach L, Morgan M, Jeffery K, Andersson M, Robinson E, Smith EG, Crook D, Peto T, Walker T. Whole-genome sequencing for predicting clarithromycin resistance in *Mycobacterium abscessus*. Antimicrob Agents Chemother. 2019. 10.1128/aac.01204-18. 30397069 10.1128/AAC.01204-18 PMC6325232

[58] Woods GL, Brown-Elliott BA, Conville PS, Desmond EP, Hall GS, Lin G, Pfyffer GE, Ridderhof JC, Siddiqi SH, Wallace RJ Jr, Warren NG, Witebsky FG. Susceptibility testing of mycobacteria, nocardiae, and other aerobic actinomycetes. Wayne: Clinical and Laboratory Standards Institute; 2011. 31339680

[59] Johansen MD, Herrmann JL, Kremer L. Non-tuberculous mycobacteria and the rise of *Mycobacterium abscessus*. Nat Rev Microbiol. 2020;18(7):392–407. 32086501 10.1038/s41579-020-0331-1

[60] Foik IP, Tuszynska I, Feder M, Purta E, Stefaniak F, Bujnicki JM. Novel inhibitors of the rRNA ErmC’ methyltransferase to block resistance to macrolides, lincosamides, streptogramine B antibiotics. Eur J Med Chem. 2018;146:60–7. 29396363 10.1016/j.ejmech.2017.11.032

[61] Fischer TR, Meidner L, Schwickert M, Weber M, Zimmermann RA, Kersten C, Schirmeister T, Helm M. Chemical biology and medicinal chemistry of RNA methyltransferases. Nucleic Acids Res. 2022;50(8):4216–45. 35412633 10.1093/nar/gkac224 PMC9071492

