# Supplementary material for: Cyclophostin and Cyclipostins analogues counteract macrolide-induced resistance mediated by erm(41) in Mycobacterium abscessus
Source: J Biomed Sci. 2024 Dec 3;31:103. doi: 10.1186/s12929-024-01091-w (PMC11613490; doi:10.1186/s12929-024-01091-w)
Supplement: Supplementary file 1 — Additional file 1. [file 12929_2024_1091_MOESM1_ESM.docx]

**SUPPLEMENTAL DATA**

**Cyclophostin and Cyclipostins analogs counteract macrolide-induced resistance mediated by Erm(41) in *Mycobacterium abscessus***

Morgane Sarrazin^1^, Isabelle Poncin^1^, Patrick Fourquet^2^, Stéphane Audebert^2^, Luc Camoin^2^, Yann Denis^3^, Pierre Santucci^1^, Christopher D. Spilling^4^, Laurent Kremer^5,6^, Vincent Le Moigne^7^, Jean‑Louis Herrmann^7,8^, Jean‑François Cavalier^1^ and Stéphane Canaan^1,§^

^1^ Aix-Marseille Univ., CNRS, LISM UMR7255, IMM-FR3479, Marseille, France

^2^ INSERM, CNRS, Institut Paoli-Calmettes, CRCM, Marseille Protéomique, Aix-Marseille Univ., France

^3^ Plateforme Transcriptome, Aix-Marseille Univ., CNRS, IMM-FR3479, Marseille, France

^4^ Department of Chemistry and Biochemistry, University of Missouri St. Louis, MO, USA

^5^ Centre National de la Recherche Scientifique UMR 9004, Institut de Recherche en Infectiologie de Montpellier (IRIM), Université de Montpellier, 34293 Montpellier, France

^6^ INSERM, Institut de Recherche en Infectiologie de Montpellier, 34293 Montpellier, France

^7^ Université Paris-Saclay, UVSQ, INSERM, Infection et Inflammation, Montigny‑Le‑Bretonneux, France

^8^ Assistance Publique-Hôpitaux de Paris, Hôpitaux Universitaires Ile-de-France Ouest, GHU Paris-Saclay, Hôpital Raymond Poincaré, Garches, France.

§ Corresponding author: [canaan@imm.cnrs.fr](mailto:canaan@imm.cnrs.fr)

**Figure S1**

**
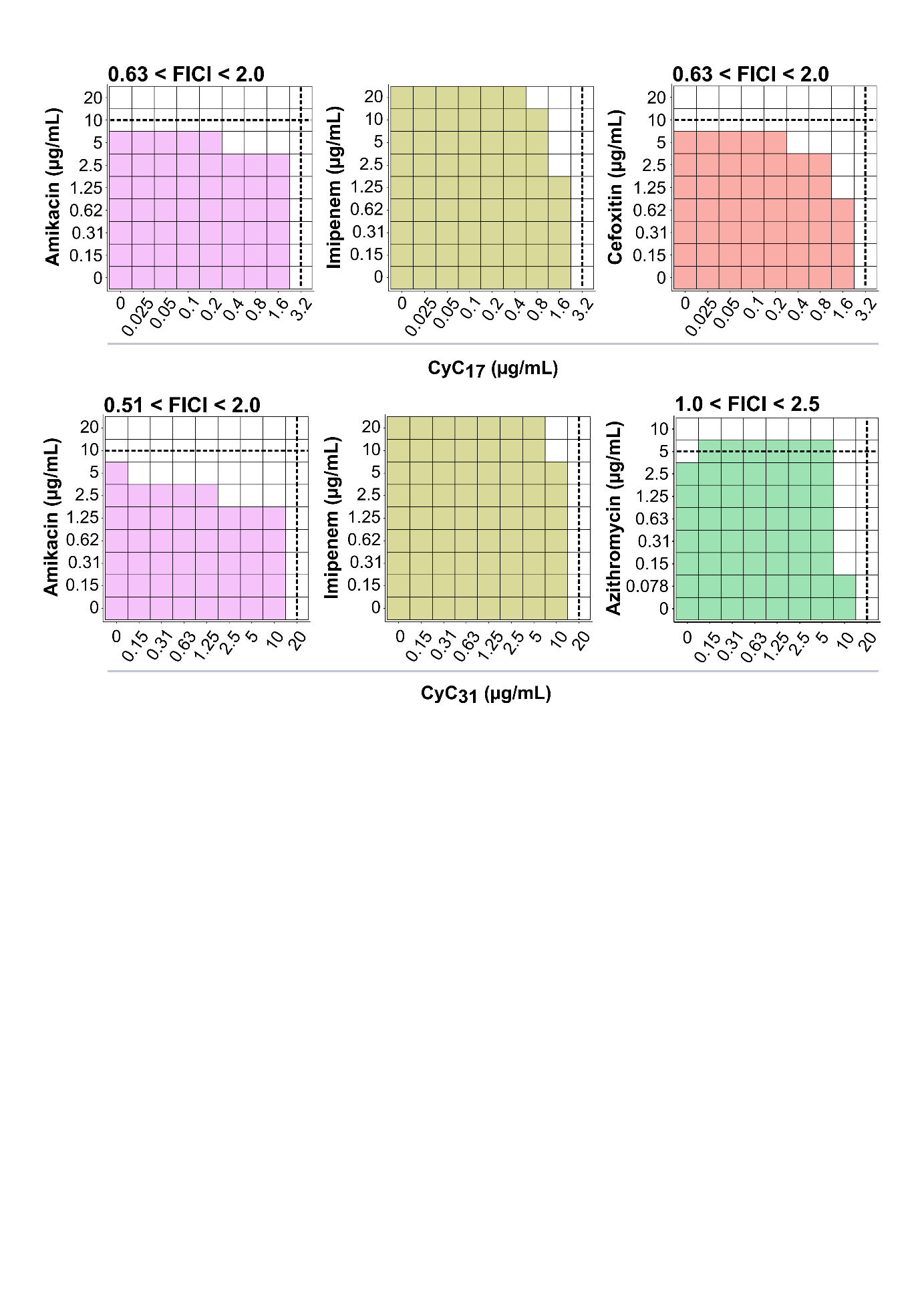
**

**Figure S1: Activity of CyC-antibiotics combination against *M. abscessus*.** Schematic checkerboard representation of association between amikacin, imipenem, or cefoxitin with **CyC_17_**; and between amikacin, imipenem, or azithromycin with **CyC_31_**. The growth is represented by the colored boxes. Data are representative of three biological replicates. Black dotted lines represent the respective MIC of the drug (horizontal) and the **CyC** (vertical). The absence of black dotted lines means that the MIC is over the concentration range. The calculated fractional inhibitory concentration index (FICI) of the best combination is indicated on top of each representation. The smallest FICI obtained for a combination is taken as the FICI of the association.

**Figure S2**

**
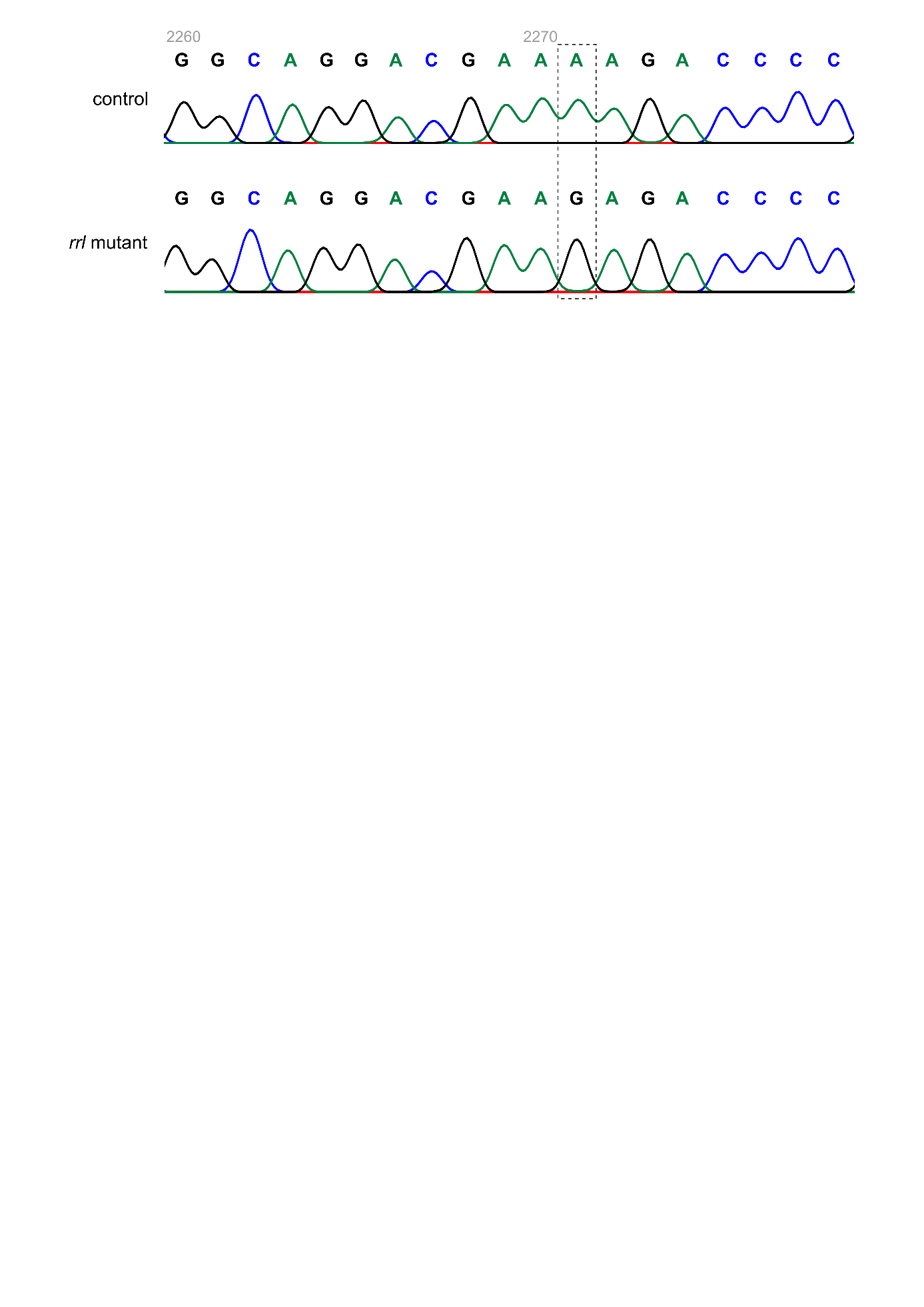
**

**Figure S2: Sequencing of the *rrl* gene coding for 23S rRNA.** DNA chromatogram of *rrl* gene between 2260 and 2278 positions for macrolide sensitive strain (up) and spontaneous mutant generated by high macrolide concentration exposure (down).

**Table S1: Mean relative growth obtained as a function of the different CyC concentrations tested for control, AZM-treated and CLR-treated conditions.**

| [**CyC_17_**]  µg/mL | 0 | MIC_/64_  0.05 | MIC_/32_  0.1 | MIC_/16_  0.2 | MIC_/8_  0.4 | MIC_/4_  0.8 | MIC_/2_  1.6 | MIC  3.2 |
| --- | --- | --- | --- | --- | --- | --- | --- | --- |
| Relative growth of all strains (%) | 100±0 | 100±12 | 75±14 | 38±11 | 31±4 | 23±4 | 18±3 | 5±5 |
| [**CyC_31_**]  µg/mL | 0 | MIC_/64_  0.31 | MIC_/32_  0.63 | MIC_/16_  1.25 | MIC_/8_  2.5 | MIC_/4_  5 | MIC_/2_  10 | MIC  20 |
| Relative growth of all strains (%) | 100±0 | 72±26 | 44±17 | 36±9 | 33±6 | 31±8 | 25±3 | 10±6 |

**Table S2: Activity of CyC-macrolides association on macrolide-resistant *M. abscessus* strains.** Values correspond to the MIC of macrolide according to the different **CyC** concentrations tested in each condition (Control, AZM-treated, CLR-treated). The fold-change was calculated relative to the MIC of macrolide without **CyC**. Each **CyC** concentration was reported relative to the **CyC**'s MIC. The MICs are given in µg/mL and were obtained from three independent biological replicates.

| **Control** | | [**CyC_17_**]  µg/mL | 0 | | MIC_/64_  0.05 | MIC_/32_  0.1 | | MIC_/16_  0.2 | MIC_/8_  0.4 | MIC_/4_  0.8 | MIC_/2_  1.6 | MIC  3.2 |
| --- | --- | --- | --- | --- | --- | --- | --- | --- | --- | --- | --- | --- |
|  |  | MIC_CLR_ | 0.6 | | 0.3 | 0.3 | | 0.3 | 0.3 | 0.3 | 0.3 | < 0.3 |
|  |  | Fold-change | - | | 2 | 2 | | 2 | 2 | 2 | 2 | > 2 |
|  |  | MIC_AZM_ | 6 | | 2 | 2 | | 2 | 0.8 | 0.8 | 0.8 | < 0.8 |
|  |  | Fold-change | - | | 3 | 3 | | 3 | 7.5 | 7.5 | 7.5 | > 7.5 |
|  |  | [**CyC_31_**]  µg/mL | 0 | | MIC_/64_  0.31 | MIC_/32_  0.63 | | MIC_/16_  1.25 | MIC_/8_  2.5 | MIC_/4_  5 | MIC_/2_  10 | MIC  20 |
|  |  | MIC_CLR_ | 0.6 | | 0.3 | 0.3 | | 0.3 | 0.3 | 0.3 | 0.3 | < 0.3 |
|  |  | Fold-change | - | | 2 | 2 | | 2 | 2 | 2 | 2 | > 2 |
|  |  | MIC_AZM_ | 6 | | 3 | 3 | | 3 | 3 | 3 | 2 | < 0.8 |
|  |  | Fold-change | - | | 2 | 2 | | 2 | 2 | 2 | 3 | > 7.5 |
| **AZM-treated** | | [**CyC_17_**]  µg/mL | 0 | | MIC_/64_  0.05 | MIC_/32_  0.1 | | MIC_/16_  0.2 | MIC_/8_  0.4 | MIC_/4_  0.8 | MIC_/2_  1.6 | MIC  3.2 |
|  |  | MIC_CLR_ | 40 | | 5 | 3 | | 5 | 3 | 1 | 0.3 | < 0.3 |
|  |  | Fold-change | - | | 8 | 13 | | 8 | 13 | 40 | 133 | > 133 |
|  |  | MIC_AZM_ | > 100 | | 25 | 50 | | 25 | 13 | 3 | 2 | < 0.8 |
|  |  | Fold-change | - | | 4 | 2 | | 4 | 7 | 33 | 50 | > 125 |
|  |  | [**CyC_31_**]  µg/mL | 0 | | MIC_/64_  0.31 | MIC_/32_  0.63 | | MIC_/16_  1.25 | MIC_/8_  2.5 | MIC_/4_  5 | MIC_/2_  10 | MIC  20 |
|  |  | MIC_CLR_ | 40 | | 5 | 5 | | 5 | 3 | 3 | 1 | < 0.6 |
|  |  | Fold-change | - | | 8 | 8 | | 8 | 13 | 13 | 40 | > 66 |
|  |  | MIC_AZM_ | > 100 | | 50 | 50 | | 50 | 50 | 50 | 13 | < 0.8 |
|  |  | Fold-change | - | | 2 | 2 | | 2 | 2 | 2 | 7 | > 125 |
| **CLR-treated** | | [**CyC_17_**]  µg/mL | 0 | | MIC_/64_  0.05 | MIC_/32_  0.1 | | MIC_/16_  0.2 | MIC_/8_  0.4 | MIC_/4_  0.8 | MIC_/2_  1.6 | MIC  3.2 |
|  |  | MIC_CLR_ | > 40 | | 40 | 20 | | 20 | 5 | 1 | 0.3 | < 0.3 |
|  |  | Fold-change | - | | - | 2 | | 2 | 8 | 40 | 133 | > 133 |
|  |  | MIC_AZM_ | > 100 | | 100 | 100 | | 100 | 100 | 50 | 3 | < 0.8 |
|  |  | Fold-change | - | | - | - | | - | - | 2 | 33 | > 125 |
|  |  | [**CyC_31_**]  µg/mL | 0 | | MIC_/64_  0.31 | MIC_/32_  0.63 | | MIC_/16_  1.25 | MIC_/8_  2.5 | MIC_/4_  5 | MIC_/2_  10 | MIC  20 |
|  |  | MIC_CLR_ | > 40 | | > 40 | > 40 | | > 40 | 20 | 3 | 0.6 | < 0.3 |
|  |  | Fold-change | - | | - | - | | - | 2 | 13 | 66 | > 133 |
|  |  | MIC_AZM_ | > 100 | | 100 | 100 | | 100 | 100 | 100 | 50 | < 0.8 |
|  |  | Fold-change | - | | - | - | | - | - | - | 2 | > 125 |
|  | | Susceptible | | | MIC ≤ 2 | | |  |  |  |  |  |
|  | | Intermediate | | | 2 < MIC < 8 | | |  |  |  |  |  |
|  | | Resistant | | | MIC ≥ 8 | | |  |  |  |  |  |

**Table S3: Activity of the CyC-macrolide combination on *rrl* mutant strain macrolides resistant of *M. abscessus*.** Values correspond to the MIC of macrolide according to the different **CyC** concentrations tested. The MIC are given in µg/mL and were obtained from three independent biological replicates.

| ***rrl* mutant** | [**CyC**] | 0 | MIC_/64_ - MIC_/2_ | MIC |
| --- | --- | --- | --- | --- |
|  | MIC_CLR_ | > 40 | > 40 | < 0.3 |
|  | MIC_AZM_ | > 100 | > 100 | < 0.8 |
